# Attack Vulnerability of Network Controllability

**DOI:** 10.1371/journal.pone.0162289

**Published:** 2016-09-02

**Authors:** Zhe-Ming Lu, Xin-Feng Li

**Affiliations:** School of Aeronautics and Astronautics, Zhejiang University, Hangzhou, P. R. China; Beihang University, CHINA

## Abstract

Controllability of complex networks has attracted much attention, and understanding the robustness of network controllability against potential attacks and failures is of practical significance. In this paper, we systematically investigate the attack vulnerability of network controllability for the canonical model networks as well as the real-world networks subject to attacks on nodes and edges. The attack strategies are selected based on degree and betweenness centralities calculated for either the initial network or the current network during the removal, among which random failure is as a comparison. It is found that the node-based strategies are often more harmful to the network controllability than the edge-based ones, and so are the recalculated strategies than their counterparts. The Barabási-Albert scale-free model, which has a highly biased structure, proves to be the most vulnerable of the tested model networks. In contrast, the Erdős-Rényi random model, which lacks structural bias, exhibits much better robustness to both node-based and edge-based attacks. We also survey the control robustness of 25 real-world networks, and the numerical results show that most real networks are control robust to random node failures, which has not been observed in the model networks. And the recalculated betweenness-based strategy is the most efficient way to harm the controllability of real-world networks. Besides, we find that the edge degree is not a good quantity to measure the importance of an edge in terms of network controllability.

## Introduction

Complex networks are ubiquitous in nature and society describing various systems [1–3], the dynamics taking place on them has become one of the most popular research fields in the past decades [4–6]. Most previous works have been focused on dynamics such as epidemic spreading [7, 8], synchronization [6, 9, 10], random walk [11, 12], opinion formation [13, 14], and so on. However, our ultimate goal of studying complex networks is to develop the capacity to control them [15]. That is, driving the network from any initial state to any desired final state in finite time [15–18]. Although great efforts [19–24] have been devoted to understanding the controllability of networks, the results are less than satisfactory. Recently, Liu et al. [15] made a significant breakthrough, they applied the structural control theory [25] to directed networks and proved that the minimal number of nodes needed to fully control a network is determined by its ‘maximum matching’ [26]. Following this seminal work, some intensive and extensive issues have been carefully addressed, such as controlling linear edge dynamics [27], effects of topological characteristics on controllability [28], energy needed for control [29], control centrality [30] and controllability optimization [31]. Besides, given the limitation that the structural controllability framework is only applicable to directed networks, Yuan et al. [32] presented the exact controllability framework to explore the controllability of arbitrary networks, especially for undirected networks and networks with exact link weights.

Most of the existing works assume that networks are in a relatively safe environment. However, the real-world networks are always confronting with random or intentional node or edge attacks. For example, in the computer networks [33], node attacking can be interpreted as breakdowns of servers by malicious hackers while edge attacking may correspond to the cutting-off of communication cables. In power grids [34], attacks on nodes can be interpreted as substation failures while attacks on edges may correspond to the cases that the connections of subsections are cut off so the power cannot be transmitted from one substation to another. Previous studies have shown that random failures and intentional attacks can easily damage network functions such as connectivity [35] and synchronization [36]. Therefore, it is natural and interesting to ask how the attacks will affect the controllability of networks. Liu et al. [15] first addressed this problem using core percolation, pointing out that the robustness of network controllability is closely related to its core. Pu et al. [37] investigated the robustness of control under node-based cascade failures and found that even if a small range of node failures can trigger great harm to the network controllability. Inspired by Pu et al.’s work, Nie et al. [38] studied edge-based cascade failures and showed that the larger scale of cascades in scale-free networks does not mean that there will be more increments of driver nodes.

In addition to cascade attacks [39], prominence based attacks, especially degree and betweenness based attacks, are the most common attacks in practice [35]. For example, it has been shown that even if 1% of the most highly connected routers were incapacitated, the average performance of Internet would drop 50% [40]. Therefore, it is of practical significance to study the robustness of network controllability subject to this kind of attacks. In this paper, we systematically investigate the attack vulnerability of network controllability for the canonical model networks as well as the real-world networks subject to degree and betweenness based attacks on nodes and edges. For each case of attacks, five different strategies are employed and the network controllability is quantitatively measured by the fraction of driver nodes. Furthermore, we also investigate the control robustness of 25 real-world networks.

The rest of the paper is organized as follows. Section II gives a brief review of network controllability. Section III introduces our methods, including definitions, attack strategies and benchmark networks. Our main results and discussions are presented in Section IV. Finally, Section V concludes the whole paper.

## Network Controllability

In this section, we briefly review the controllability of complex networks, both the structural controllability framework [15] and the exact controllability framework [32] are introduced. The former is only applicable to directed networks whereas the latter can treat arbitrary networks without any limitations [32].

### Structural Controllability

Consider a network of *N* nodes governed by the following linear time invariant dynamics [15]:

$$\begin{array}{c} \dot{x}\left(t\right)=Ax\left(t\right)+Bu\left(t\right) \end{array}$$ (1)

where ***x***(*t*) = (*x*_1_(*t*), …, *x*_*N*_(*t*))^*T*^ stands for the states of nodes at time *t*, the *N* × *N* matrix *A* denotes the coupling strength between *N* nodes, where the element *a*_*ij*_ gives the strength or weight that node-*j* can affect node-*i*. ***u***(*t*) = (*u*_1_(*t*), *u*_2_(*t*), …, *u*_*M*_(*t*))^*T*^ is the vector of input signals and the *N* × *M* (*M* ≤ *N*) matrix *B* is called input matrix which defines how input signals are connected to the nodes in the network, which are often called ‘driver nodes’.

According to the classic Kalman rank condition [16, 41], the system described by Eq (1) is controllable if and only if

$$\begin{array}{c} \text{rank}\left(C\right)=\text{rank}(\left[B,AB,A^{2}B,\ldots ,A^{N-1}B\right])=N \end{array}$$ (2)

where *C* = [*B*, *AB*, *A*^2^*B*, …, *A*^*N* − 1^*B*] is called controllability matrix. Since the matrix *A* is fixed for a given network, in order to make the network fully controllable, one need to choose a suitable matrix *B* having the minimal number of driver nodes to satisfy the Kalman rank condition. However, the practical difficulty lies in that there are 2^*N*^ − 1 possible combinations of selecting driver nodes. To say the least, even one can enumerate all the combinations efficiently, the link weights (*a*_*ij*_) are often not known for real-world networks. In order to overcome the inherently incomplete information of link weights, Liu et al. [15] introduced the concept of ‘structural controllability’ [25] to complex networks. The structural controllability can ensure that a network is controllable for almost all weight combinations except for some pathological cases [15]. The authors also developed the minimum input theory to avoid brute-force searching, which states that the minimal number of driver nodes needed to fully control a network, *N*_*D*_, is determined by the maximum matching [26] in the network [15], where the unmatched nodes are exactly driver nodes. This theory allows one to find the driver nodes within $O(\sqrt{N}L)$ rather than *O*(2^*N*^) time, where *L* denotes the number of links [15].

Liu et al.’s work also showed that a network’s controllability is mainly determined by its underlying degree distribution, networks that are sparse and heterogeneous are more difficult to control [15]. Let *n*_*D*_ = *N*_*D*_/*N* denote the density of driver nodes, for Erdős-Rényi (ER) networks [42] with mean degree 〈*k*〉, Liu et al. give *n*_*D*_ analytically as [15]

$$\begin{array}{c} n_{D}\approx e^{-\frac{\left\langle k\right\rangle }{2}} \end{array}$$ (3)

While for scale-free networks [2] with mean degree 〈*k*〉 and degree exponent *γ* = *γ*_*in*_ = *γ*_*out*_, *n*_*D*_ is given by [15]

$$\begin{array}{c} n_{D}\approx \text{exp}[-\frac{1}{2}(1-\frac{1}{\gamma -1})\left\langle k\right\rangle ] \end{array}$$ (4)

### Exact Controllability

The exact controllability framework was proposed by Yuan et al. [32] to overcome the limitation that the structural controllability framework can only treat directed networks. As an alternative, the new paradigm can be applied to arbitrary network structures and link weights without any limitations [32].

Instead of empolying the Kalman rank condition, the exact controllability framework is based on the equivalent Popov-Belevitch-Hautus (PBH) rank condition [43], which stipulates that the system (Eq (1)) is controllable, if and only if

$$\begin{array}{c} \text{rank}(\psi I_{N}-A,B)=N \end{array}$$ (5)

holds for any complex number *ψ*, where *I*_*N*_ is the *N* × *N* identify matrix. It can be further proved that full control can be guaranteed if and only if all the eigenvalues *λ* of *A* satisfy Eq (5) [32]. From the perspective of matrix, the minimal number of driver nodes, *N*_*D*_, is defined by matrix *B* with *N*_*D*_ = min{rank(*B*)}. Equivalently, Yuan et al. proved that for any network with arbitrary matrix *A*, *N*_*D*_ is actually determined by the maximum geometric multiplicity *μ*(*λ*_*i*_) of the eigenvalue *λ*_*i*_ of *A* [32], i.e.

$$\begin{array}{c} N_{D}=\max_{i}\left\{\mu \left(\lambda_{i}\right)\right\} \end{array}$$ (6)

where *λ*_*i*_(*i* = 1, …, *l*) is the nonidentical eigenvalues of *A* and *μ*(*λ*_*i*_) = *N*−rank(*λ*_*i*_*I*_*N*_−*A*) is the geometric multiplicity of *λ*_*i*_. In particular, for undirected networks, *N*_*D*_ is simply determined by the maximum algebraic multiplicity *δ*(*λ*_*i*_) of *λ*_*i*_ [32], i.e.

$$\begin{array}{c} N_{D}=\max_{i}\left\{\delta \left(\lambda_{i}\right)\right\} \end{array}$$ (7)

For large sparse networks, *N*_*D*_ can be dramatically simplified in terms of rank(*A*) [32], i.e.

$$\begin{array}{c} N_{D}=\max\left\{1,N-\text{rank}\left(A\right)\right\} \end{array}$$ (8)

## Methods

In this section, we introduce our experimental methods, which include definitions, attack strategies and benchmark networks.

### Definitions

In this paper, we allow the existence of self loops in the network but multiple (parallel) edges are not allowed. We also denote the number of nodes as *N* and the number of edges as *L*.

The measure of controllability *n*_*D*_, or simply controllability, of a network is defined as the ratio of the minimum number of driver nodes *N*_*D*_ to the network size *N* [15], i.e.

$$\begin{array}{c} n_{D}=N_{D}/N \end{array}$$ (9)

The degree *k*_*v*_ of a node *v* is defined as the number of its direct connections to other nodes. The degree *k*_*e*_ of an edge *e* is defined as

$$\begin{array}{c} k_{e}=k_{u}k_{v} \end{array}$$ (10)

where *e* connects nodes *u* and *v* with degrees *k*_*u*_ and *k*_*v*_.

The betweenness centrality of a node *v* is defined as

$$\begin{array}{c} C_{B}\left(v\right)=\sum_{s\neq v\neq t\in V}\frac{\sigma_{st}\left(v\right)}{\sigma_{st}} \end{array}$$ (11)

where *σ*_*st*_ is the total number of shortest paths from node *s* to node *t* and *σ*_*st*_(*v*) is the number of those paths that pass through *v*. Similarly, the edge betweenness centrality of an edge *e* is defined as

$$\begin{array}{c} C_{B}\left(e\right)=\sum_{s\neq t\in V}\frac{\sigma_{st}\left(e\right)}{\sigma_{st}} \end{array}$$ (12)

where *σ*_*st*_(*e*) is the number of shortest paths from node *s* to node *t* that include the edge *e*. Throughout the present paper, we call *C*_*B*_(*v*) and *C*_*B*_(*e*) the node betweenness and the edge betweenness for brevity.

### Attack Strategies

Here we consider five different attack strategies for nodes and edges. The nodes are removed in the descending order of degree or betweenness centrality calculated for either the initial network or the current network during the removal procedure. Random attack (also called random failure) is taken as a comparison. The five node attack strategies are described as follows:

- Random Attack (RA): to remove node one by one randomly.
- Initial Degree Attack (ID): to remove node one by one in the descending order of degree using the degree distribution of the initial network.
- Initial Betweenness Attack (IB): to remove node one by one in the descending order of betweenness using the betweenness distribution of the initial network.
- Recalculated Degree Attack (RD): to remove node one by one in the descending order of degree using the recalculated degree distribution at every removal step.
- Recalculated Betweenness Attack (RB): to remove node one by one in the descending order of betweenness using the recalculated betweenness distribution at every removal step.

Note that the degree-based attacks (ID and RD) are local strategies, whereas the betweenness-based attacks (IB and RB) are global strategies. Another significant difference is that the former concentrate on reducing the total edges as fast as possible whereas the latter concentrate on destroying as many as the shortest paths as possible.

The above strategies can also be applied to edge attacks by simply replacing node degree and betweenness with edge degree and betweenness. The attack vulnerability of network controllability subject to such edge attacks is also investigated in this paper.

### Networks

We select four canonical model networks as well as two real networks as benchmarks to study the robustness of network controllability. All the six networks are undirected and in the following experiments the exact controllability framework [32] will be adopted to calculate their controllability.

The ER model [42] is a classic random network with a Poisson-type degree distribution *P*(*k*) = *e*^−〈*k*〉^〈*k*〉^*k*^/*k*!, a logarithmically increasing average path length and a small clustering coefficient close to zero. The Watts-Strogatz (WS) model [1] and Newman-Watts (NW) model [44] are classic small world networks, which have short average path lengths (∼ln *N*) and high clustering coefficients. Both of them are constructed from a regular ring lattice with each node linking to its left and right *K*/2 nearest neighbors, the only difference is that the former rewires edges with probability *p* whereas the latter adds edges with probability *p*. The shape of their degree distribution is similar to that of ER, having a pronounced peak at *k* = *K* and decaying exponentially for large |*k* − *K*|. The Barabási-Albert (BA) model [2] is a representative scale free network with the power-law degree distribution *P*(*k*)∼*k*^ − 3^.

**Fig 1:**
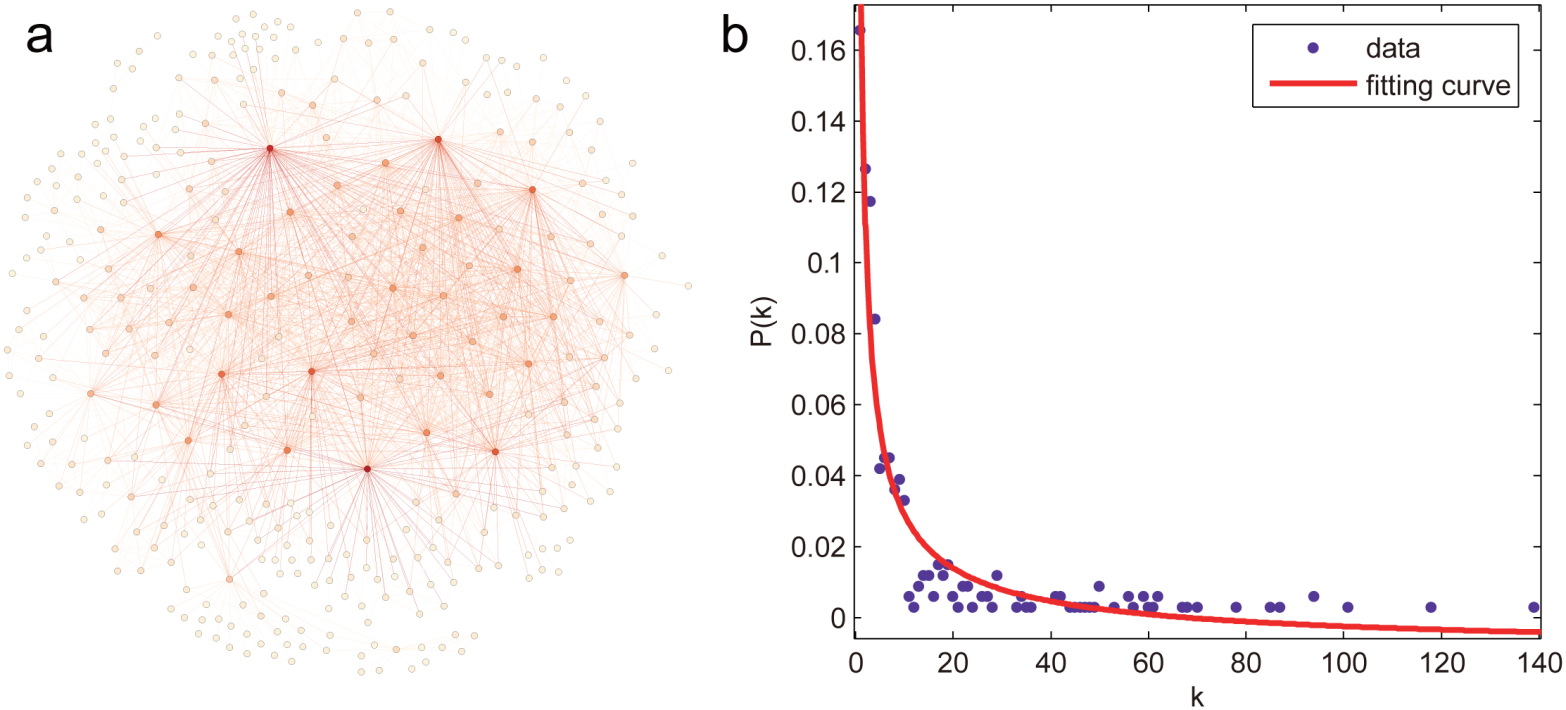
The USAir97 network. (a) Its topology, in which the darkness of node color is proportional to its degree. (b) Its degree distribution, where the points represent the frequency distribution of degree, the red line denotes the fitting curve with equation *P*(*k*) = 0.1948*k*^−0.6959^−0.01029, the goodness of fit is 0.9198.

The two real-world networks are the USAir97 network and the Erdos971 network. The USAir97 [45] is a network formed by the direct air route between the American airports in 1997. It has 332 nodes and 2126 edges as shown in Fig 1(a). Each node stands for an airport and each directed edge represents an airline from one airport to another. The weight of an edge represents the number of seats available on the scheduled flights. USAir97 exhibits an approximate power-law degree distribution as shown in Fig 1(b). The airports with less connections to/from other airports follow more closely with the fitting curve whereas the airports with more connections show a slight deviation.

**Fig 2:**
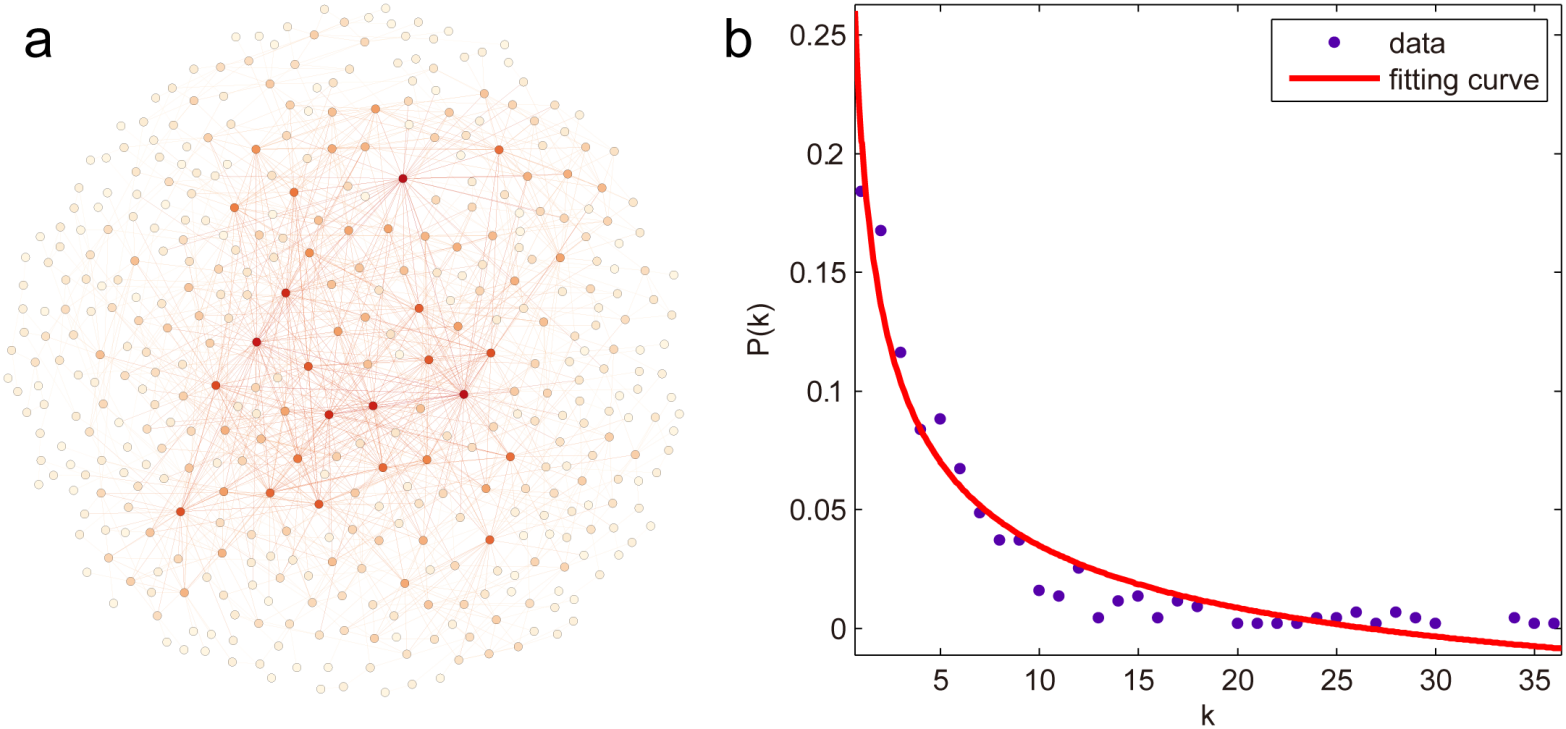
The Erdos971 network. (a) Its topology, in which the darkness of node color is proportional to its degree. (b) Its degree distribution, where the points represent the frequency distribution of degree, the red line denotes the fitting curve with equation *P*(*k*) = 0.2727*k*^−0.4256^−0.06745, the goodness of fit is 0.9434.

The Erdos971 [45] is a scientific collaboration network where each node represents an scientist who co-authored at least one paper with Paul Erdős [42], and two scientists are joined by an edge if they co-authored a paper. Note that the node corresponding to Paul Erdős himself and all the isolated components have been removed throughout the paper, the truncated network contains 429 nodes and 1312 edges. Its topology and degree distribution are displayed in Fig 2(a) and 2(b), respectively.

The parameter settings and summaries of the benchmark networks are shown in Table 1.

**Table 1:** The summaries of benchmark networks used in this paper.

Type	Network	*N*	*L*	〈*k*〉
Random Network	ER	200	400	4.0
Small World	WS	200	400	4.0
Small World	NW	200	440	4.4
Scale Free	BA	200	591	5.91
Transportation	USAir97	332	2126	6.4036
Scientific Collaboration	Erdos971	429	1312	6.1166

All the networks are undirected and for each network, we show its type, name, number of nodes (*N*), number of edges (*L*), and average degree (〈*k*〉). Note that the original Erdos971 network has 472 nodes and 1314 edges, here we only keep its largest connected component.

## Results and Discussions

### Node Attack

**Fig 3:**
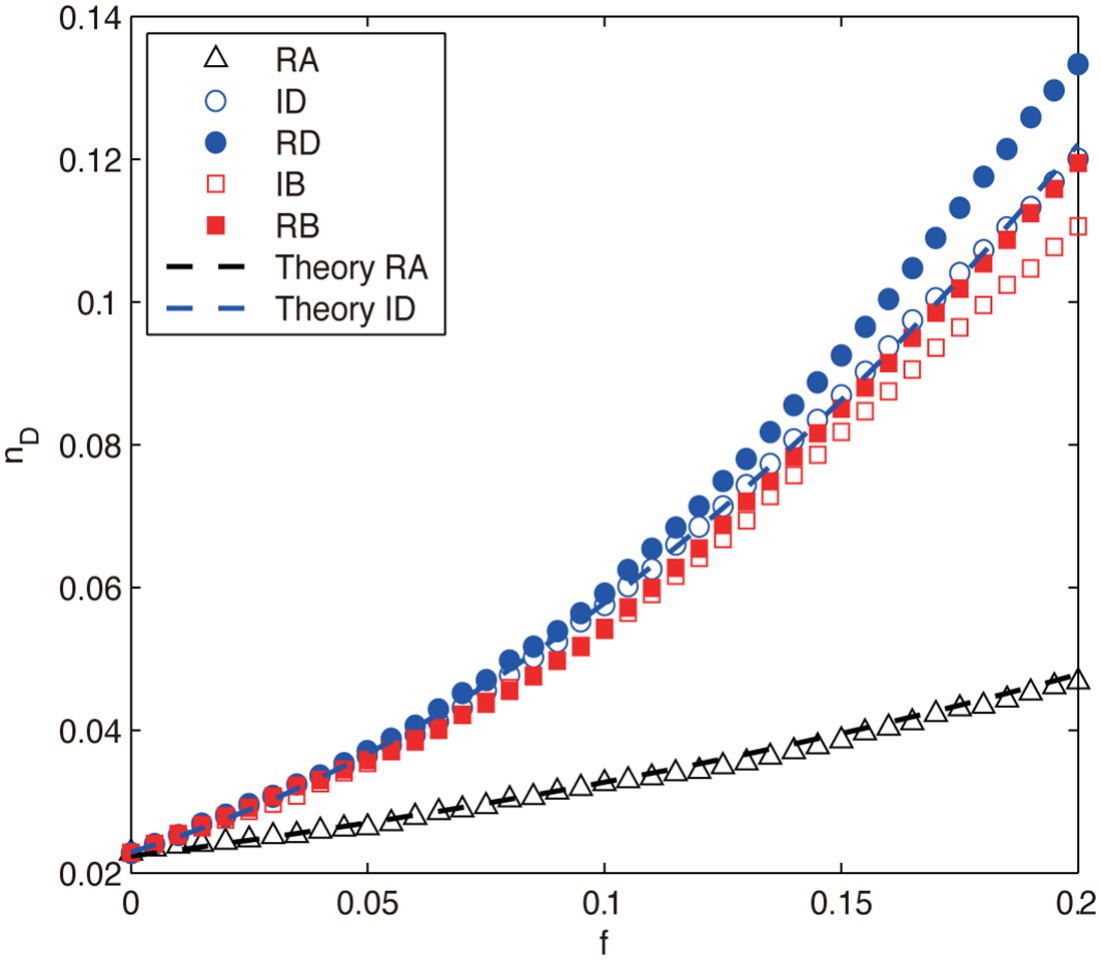
The density of driver nodes *n*_*D*_ as a function of removal fraction *f* for ER network under different node attacks. The black and blue dashed lines are theoretical results obtained from Eqs (24) and (26), respectively. The numerical results are averaged over 100 independent realizations.

Firstly, we investigate the control robustness of the ER random network under node attacks and show the results in Fig 3. It can be seen that for all the attacks, *n*_*D*_ increases with the removal fraction *f*, indicating that we need constantly increase driver nodes to fully control the network, i.e., the network’s controllability is decreasing. The intentional attacks (ID, RD, IB and RB) are much more harmful than RA as their *n*_*D*_ increases much faster. For the former attacks, the difference among them is not significant in the early stage of removals (f≲0.07), which can be attributed to the high degree-betweenness correlation (high degree nodes tend to have high betweenness) [35] and the negligible redistribution of degree and betweenness information for small *f*. However, as the removals proceed (*f* > 0.07), they gradually separate by harming the network controllability in the order RD > RB ≃ ID > IB (the inequality RD > RB means that RD is more harmful than RB), revealing that degree-based attacks are more harmful than betweenness-based attacks (RD > RB and ID > IB) and the attacks based on recalculated information are, as expected, more harmful than their counterparts based on initial information (RD > ID and RB > IB), which confirms the previous conclusions that network controllability is mainly determined by degree distribution [15] but distance based measures such as betweenness can also affect the controllability [46].

For RA and ID attacks, we can give the analytical results of *n*_*D*_ by employing the cavity method [15]. The ER network follows a Possion degree distribution *P*(*k*) = *e*^−〈*k*〉^〈*k*〉^*k*^/*k*!, indicating that most nodes have almost the same degree close to 〈*k*〉^0^, the average degree of the original network. Therefore, for RA it is reasonable to assume that every removed node has degree 〈*k*〉^0^, then the average degree after removals ${\langle k\rangle }_{\text{RA}}^{{}^{\prime }}$ is

$$\begin{array}{c} {\left\langle k\right\rangle }_{\text{RA}}^{{}^{\prime }}={\left\langle k\right\rangle }^{0}-f{\left\langle k\right\rangle }^{0}=\left(1-f\right){\left\langle k\right\rangle }^{0} \end{array}$$ (13)

According to Liu et al.’s work [15], for networks where in-degrees and out-degrees share the similar distribution, *n*_*D*_ can be obtained by

$$\begin{array}{c} n_{D}=G\left(w_{2}\right)+G\left(1-w_{1}\right)-1+\left\langle k\right\rangle w_{1}\left(1-w_{2}\right) \end{array}$$ (14)

where $G\left(x\right)=\sum_{k=0}^{\infty }P\left(k\right)x^{k}$ is the predefined generating function, *w*_1_ and *w*_2_ can be derived from the following equations:

$$\begin{array}{c} w_{1}=H\left[1-H\left(1-w_{1}\right)\right] \end{array}$$ (15)

$$\begin{array}{c} w_{2}=1-H\left[1-H\left(w_{2}\right)\right] \end{array}$$ (16)

where $H\left(x\right)=\sum_{k=0}^{\infty }Q\left(k+1\right)x^{k}$ with *Q*(*k*) = *kP*(*k*)/〈*k*〉 [31].

For RA, since the removal fraction is small (*f* ≤ 0.2), we assume that the degree distribution *P*(*k*) is not significantly affected. By plugging ${\langle k\rangle }_{\text{RA}}^{{}^{\prime }}$ into *P*(*k*) and *P*(*k*) into *G*(*x*), one has

$$\begin{array}{c} G\left(x\right)=\sum_{k=0}^{\infty }\frac{e^{-{\left\langle k\right\rangle }^{0}\left(1-f\right)}{\left[{\left\langle k\right\rangle }^{0}\left(1-f\right)\right]}^{k}}{k!}x^{k}=e^{-{\left\langle k\right\rangle }^{0}\left(1-f\right)\left(1-x\right)} \end{array}$$ (17)

Similarly, by plugging *P*(*k*) into *Q*(*k*) and *Q*(*k*) into *H*(*x*), one has

$$\begin{array}{c} H\left(x\right)=\sum_{k=0}^{\infty }\frac{e^{-{\left\langle k\right\rangle }^{0}\left(1-f\right)}{\left[{\left\langle k\right\rangle }^{0}\left(1-f\right)\right]}^{k}}{k!}x^{k}=e^{-{\left\langle k\right\rangle }^{0}\left(1-f\right)\left(1-x\right)} \end{array}$$ (18)

From Eqs (15) and (16) and the monotonicity of *H*(*x*), one can easily check that *w*_1_ = *H*(*w*_2_) and *w*_2_ = 1 − *H*(1 − *w*_1_). Substituting *H*(*x*) gives

$$\begin{array}{c} w_{1}=e^{-{\left\langle k\right\rangle }^{0}\left(1-f\right)\left[H\left(1-w_{1}\right)\right]}=e^{-{\left\langle k\right\rangle }^{0}\left(1-f\right)\left[1-w_{2}\right]} \end{array}$$ (19)

$$\begin{array}{c} w_{2}=1-e^{-{\left\langle k\right\rangle }^{0}\left(1-f\right)H\left(w_{2}\right)}=1-e^{-{\left\langle k\right\rangle }^{0}\left(1-f\right)w_{1}} \end{array}$$ (20)

where *w*_1_ can be further reduced by substituting *w*_2_ into Eq (19), as follows

$$\begin{array}{c} w_{1}=\exp\left[-{\left\langle k\right\rangle }^{0}\left(1-f\right)e^{-{\left\langle k\right\rangle }^{0}\left(1-f\right)w_{1}}\right] \end{array}$$ (21)

Now *n*_*D*_ can be simplified as

$$\begin{array}{ccc} n_{D} & = & G\left(w_{2}\right)+G\left(1-w_{1}\right)-1+\left\langle k\right\rangle w_{1}\left(1-w_{2}\right)\\ & = & e^{-{\left\langle k\right\rangle }^{0}\left(1-f\right)\left[1-w_{2}\right]}+e^{-{\left\langle k\right\rangle }^{0}\left(1-f\right)w_{1}}-1+{\left\langle k\right\rangle }^{0}\left(1-f\right)w_{1}\left(1-w_{2}\right)\\ & = & w_{1}-w_{2}+{\left\langle k\right\rangle }^{0}\left(1-f\right)w_{1}\left(1-w_{2}\right) \end{array}$$ (22)

For *k* ≫ 1, one has *w*_1_ ∼ *e*^−〈*k*〉^0^(1 − *f*)^, $w_{2}=1-e^{-{\langle k\rangle }^{0}\left(1-f\right)w_{1}}∼{\langle k\rangle }^{0}\left(1-f\right)w_{1}$, and thus

$$\begin{array}{c} n_{D}∼\exp\left[-{\left\langle k\right\rangle }^{0}\left(1-f\right)\right]-{\left({\left\langle k\right\rangle }^{0}\left(1-f\right)\right)}^{2}\exp\left[-2{\left\langle k\right\rangle }^{0}\left(1-f\right)\right] \end{array}$$ (23)

Ignoring the higher order term in Eq (23) gives the final *n*_*D*_ as

$$\begin{array}{c} n_{D}∼\exp\left[-{\left\langle k\right\rangle }^{0}\left(1-f\right)\right] \end{array}$$ (24)

For ID attack, let *k*^max^ denote the maximum degree after removing *Nf* high degree nodes, then $f=\int_{k^{\max}}^{\infty }P\left(k\right)dk$. The average degree after removals, ${\langle k\rangle }_{\text{ID}}^{{}^{\prime }}$, can be derived as follows

$$\begin{array}{ccc} {\left\langle k\right\rangle }_{\text{ID}}^{{}^{\prime }} & = & \int_{0}^{k^{\max}}kP\left(k\right)dk=\int_{0}^{k^{\max}}\frac{e^{-{\left\langle k\right\rangle }^{0}}{\left({\left\langle k\right\rangle }^{0}\right)}^{k}}{(k-1)!}dk\\ & = & {\left\langle k\right\rangle }^{0}\int_{0}^{k^{\max}}\frac{e^{-{\left\langle k\right\rangle }^{0}}{\left({\left\langle k\right\rangle }^{0}\right)}^{k-1}}{(k-1)!}d\left(k-1\right)\\ & = & {\left\langle k\right\rangle }^{0}\int_{-1}^{k^{\max}-1}\frac{e^{-{\left\langle k\right\rangle }^{0}}{\left({\left\langle k\right\rangle }^{0}\right)}^{k}}{k!}dk={\left\langle k\right\rangle }^{0}\int_{0}^{k^{\max}-1}\frac{e^{-{\left\langle k\right\rangle }^{0}}{\left({\left\langle k\right\rangle }^{0}\right)}^{k}}{k!}dk\\ & = & {\left\langle k\right\rangle }^{0}(\int_{0}^{k^{\max}}\frac{e^{-{\left\langle k\right\rangle }^{0}}{\left({\left\langle k\right\rangle }^{0}\right)}^{k}}{k!}dk-\int_{k^{\max}-1}^{k^{\max}}\frac{e^{-{\left\langle k\right\rangle }^{0}}{\left({\left\langle k\right\rangle }^{0}\right)}^{k}}{k!}dk)\\ & = & {\left\langle k\right\rangle }^{0}(\int_{0}^{k^{\max}}P\left(k\right)dk-\int_{k^{\max}-1}^{k^{\max}}P\left(k\right)dk)\\ & = & {\left\langle k\right\rangle }^{0}(1-f-\int_{k^{\max}-1}^{k^{\max}}P\left(k\right)dk) \end{array}$$ (25)

For simplicity, let $\int_{k^{\max}-1}^{k^{\max}}P\left(k\right)dk=\alpha f$ where *α* is a constant coefficient, we have ${\langle k\rangle }_{\text{ID}}^{{}^{\prime }}={\langle k\rangle }^{0}\left(1-\left(1+\alpha \right)f\right)$. Using the similar method, *n*_*D*_ can be approximated as

$$\begin{array}{c} n_{D}∼\exp\left[-{\left\langle k\right\rangle }^{0}\left(1-\left(1+\alpha \right)f\right)\right] \end{array}$$ (26)

where the specific *α* can be obtained by curve fitting. From Fig 3 we can see that the theoretical results of Eqs (24) and (26) agree well with their corresponding numerical results.

**Fig 4:**
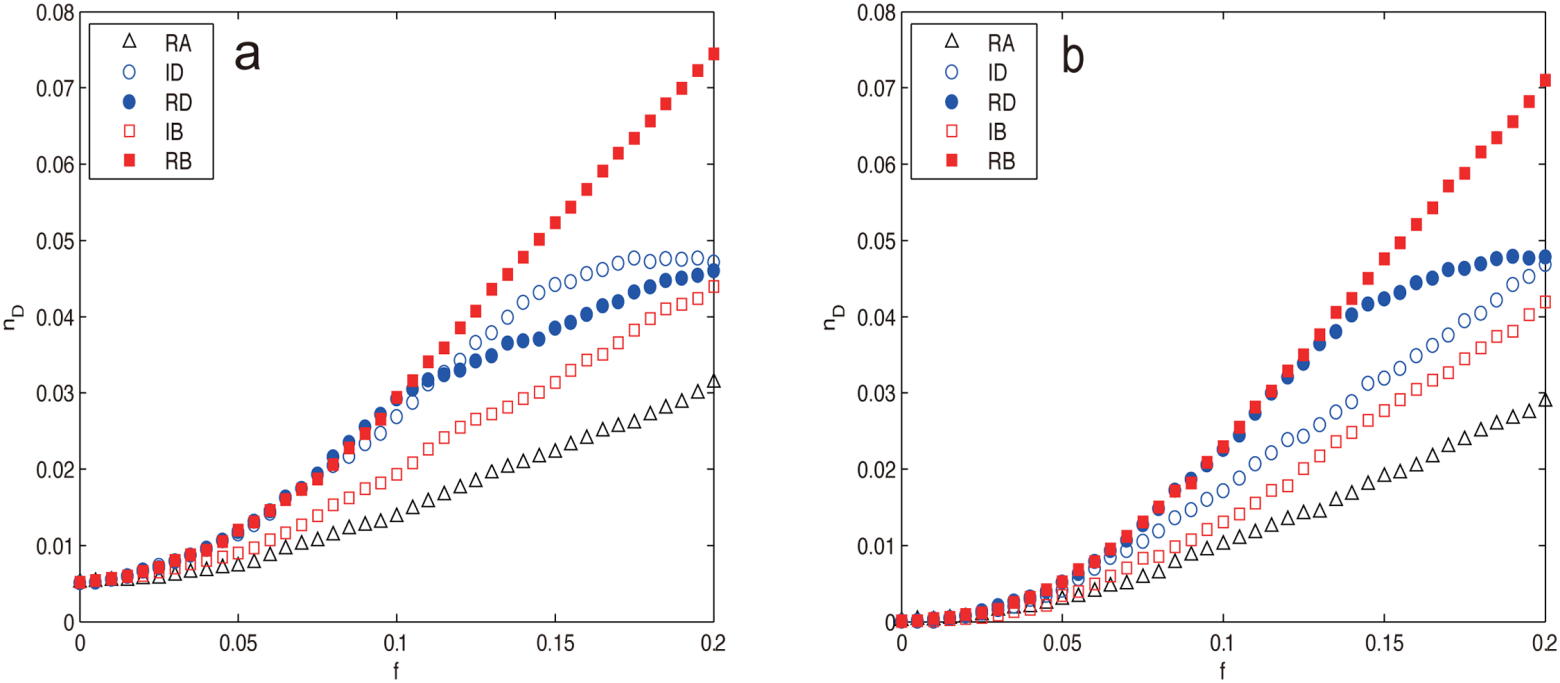
*n*_*D*_ as a function of the removal fraction *f* under different node attacks for (a) WS network and (b) NW network. The results are averaged over 100 independent realizations.

Next, we compare the two small world model networks, WS and NW, both of which have exponential cutoffs in the degree distribution. From Fig 4, we can see that the two networks exhibit very similar vulnerability behavior: *n*_*D*_ increases as the removals proceed, RB harm the controllability most, followed by the degree-base attacks and IB, RA is the least harmful strategy (RB > ID, RD > IB > RA). This phenomenon is mainly due to their similar construction method that both are generated from the one dimensional regular ring lattice. The most interesting and unexpected behavior is that for both networks, RB proves to be the most harmful strategy and the superiority even becomes more obvious for large *f* (*f* > 0.1 for WS, f≳0.15 for NW), which is beyond our expectation that degree-based attacks (at least RD) should be more harmful than betweenness-based ones due to the decisive role of degree distribution to network controllability.

**Fig 5:**
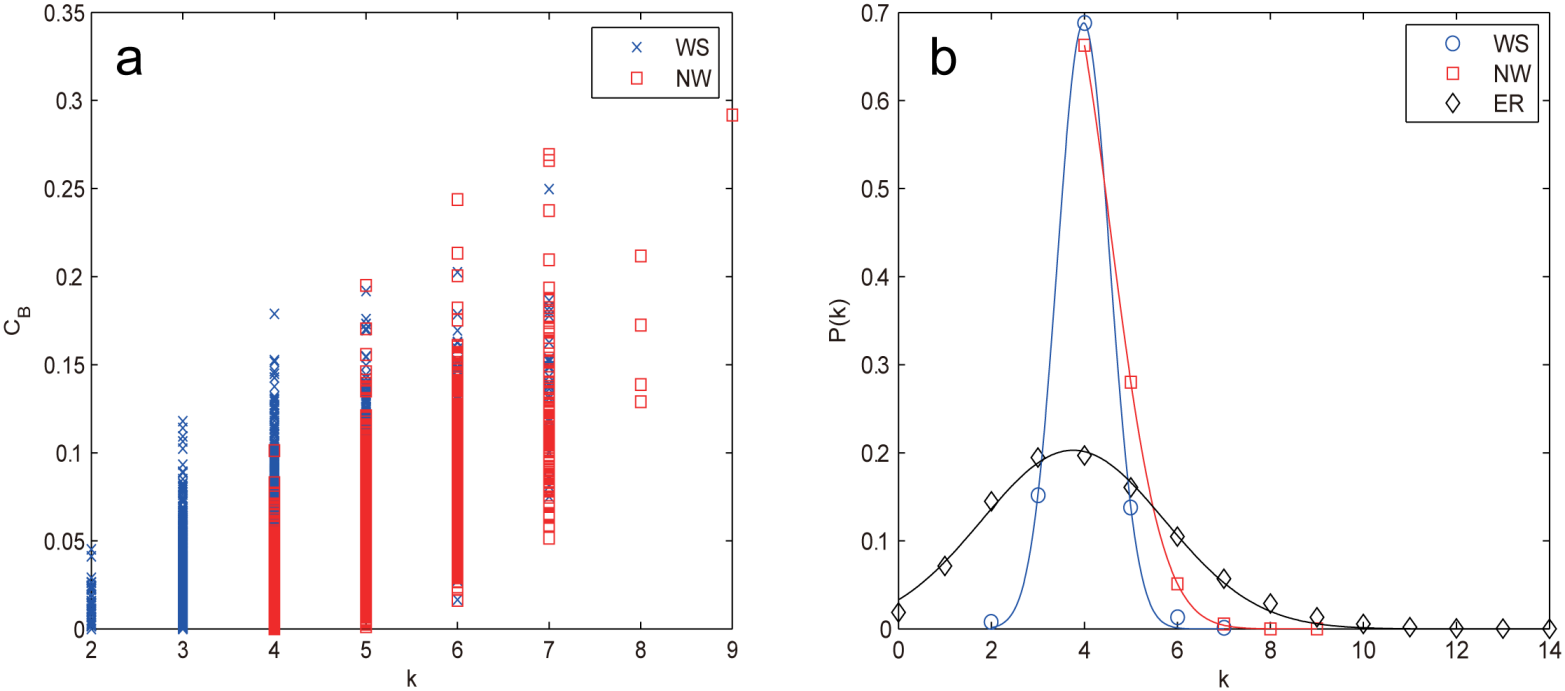
(a) Correlation between the node betweenness *C*_*B*_ and the node degree *k* for WS and NW networks. (b) The degree distribution of WS, NW and ER network.

To explain this behavior, we explore the correlation between the node betweenness *C*_*B*_ and the node degree *k* as shown in Fig 5a, from which we can see that the correlation is quite weak, which excludes the possibility that degree helps to contribute the effects of RB. Recall that the two prominent characteristics of small world networks are the short average path length (APL) and the high clustering. Since recent study [28] has shown that clustering has no discernible impact on network controllability, thus the effectiveness of RB can only be attributed to the small APL, which again confirms that betweenness also affects network’s controllability [46]. Our results clearly show that RB is the most efficient way to destroy the controllability of small world networks rather than RD.

From Fig 4, we can also see that the degree-based attacks on WS and NW networks behave quite differently from those on the ER network. The *n*_*D*_ value of the former has a clear slowdown trend for large *f* whereas the latter does not. For WS network, both RD and ID harm the network controllability equally in the interval 0 ≤ *f* ≤ *p*, after that ID prevails RD but with less and less superiority, finally coincides with the latter at *f* = 0.2. The emergence of watershed (*f* ≈ *p* = 0.1) can be explained since when *f* exceeds *p*, the original WS topology is lost, the network degenerates into a regular ring lattice. It is worth noting that WS is the only case where a procedure based on recalculated information is less harmful than its counterpart based on the initial configuration (RD < ID). For NW network, RD retains as harmful as RB for f≲0.14, then slows down for *f* > 0.14, the emergence of the turning point (*f* ≈ 0.14) occurs later than that of WS (*f* ≈ 0.1) due to its longer tail of degree distribution as shown in Fig 5b resulting from adding instead of rewiring edges.

**Fig 6:**
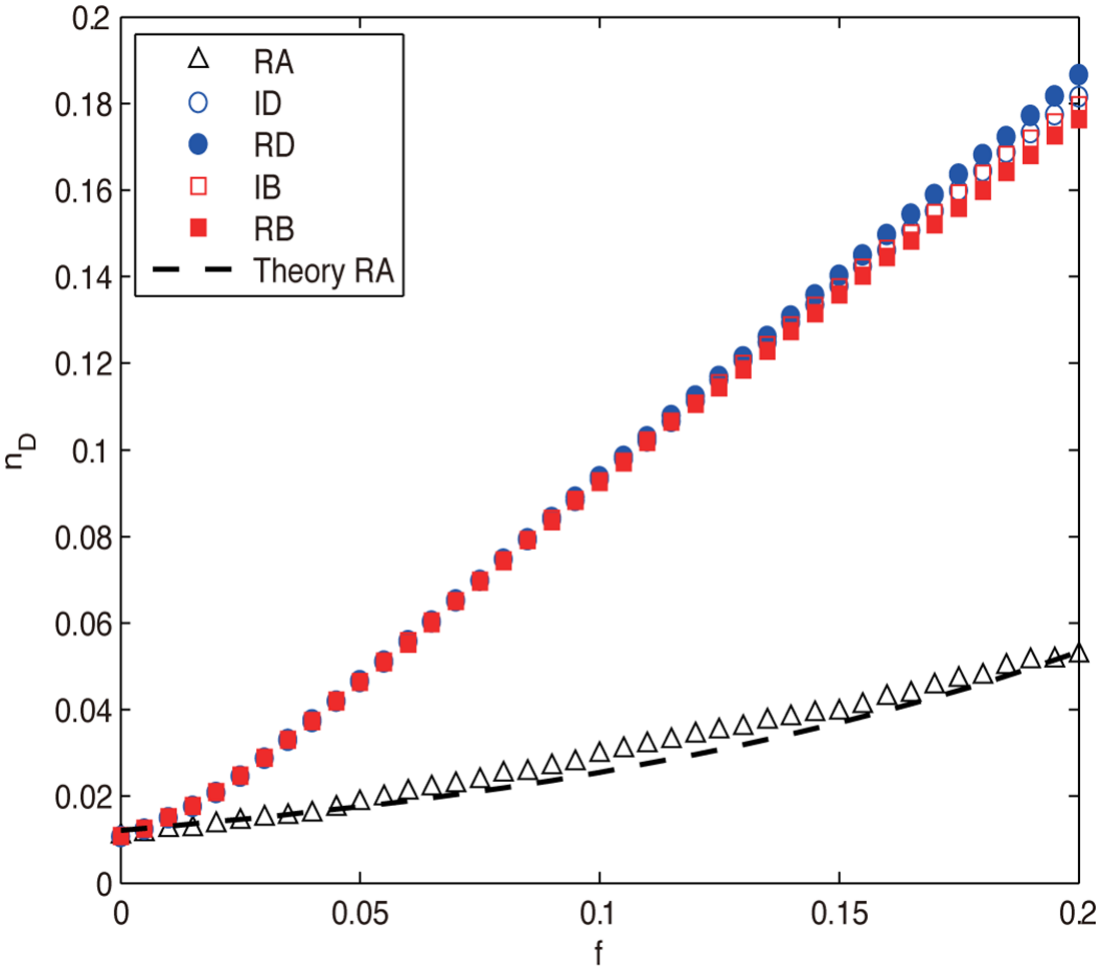
*n*_*D*_ as a function of removal fraction *f* under different node attacks for the BA scale-free network. The simulation results are averaged over 100 independent realizations, the analytical result of RA is obtained by Eq (27).

**Fig 7:**
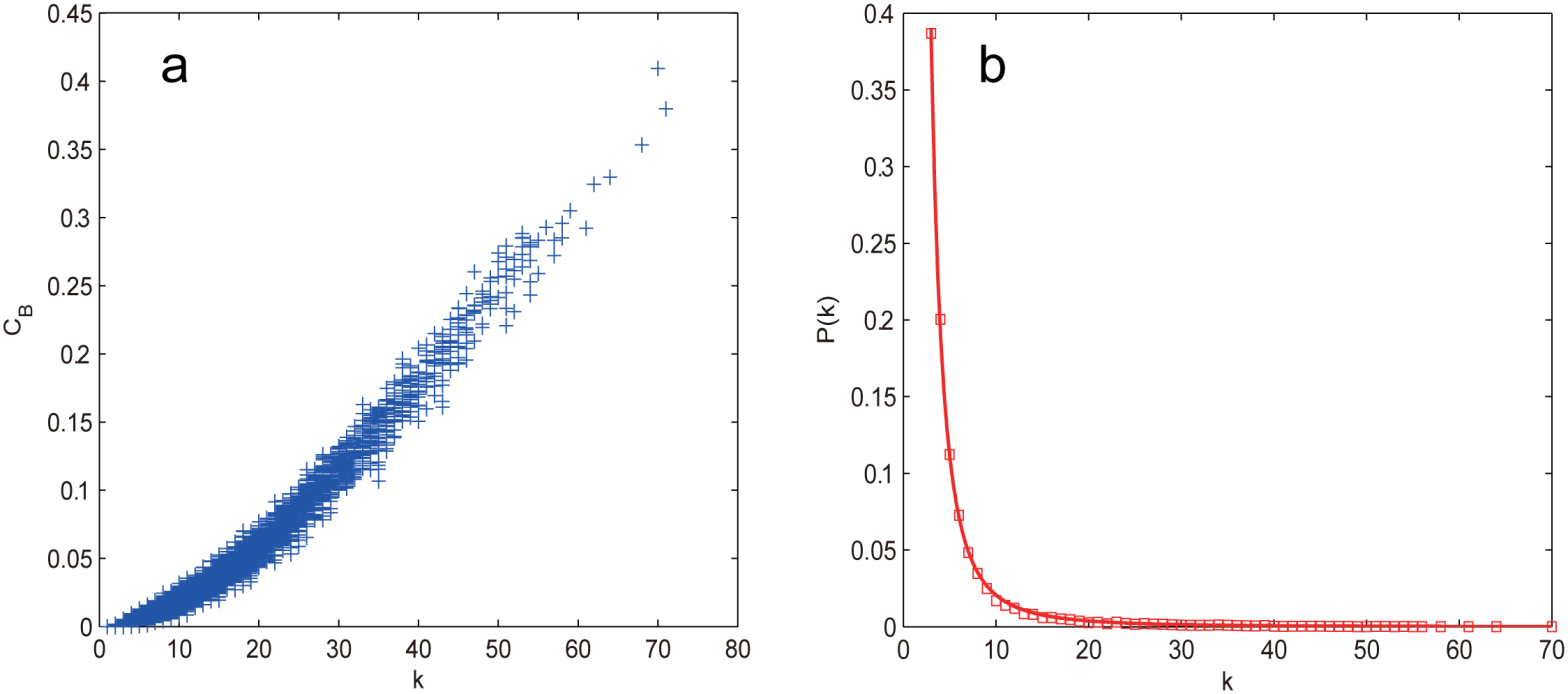
(a) Node betweenness-degree correlation of the BA network. (b) The degree distribution of the BA network.

The BA model is in focus in the first study of the control vulnerability of scale-free networks. From Fig 6, we can see that all the attacks except RA harm the network controllability equally for f≲0.15, after that the degree-based strategies prevail the betweenness-based ones with negligible advantages. This coincidence should not be attributed to the short APL like the WS and NW network but the high correlation between the node betweenness and degree as shown in Fig 7a. It can also be seen that *n*_*D*_ rises from 0.01 to about 0.19 (Δ*n*_*D*_ ≈ 0.18), which is much more significant than that of the ER (Δ*n*_*D*_ ≈ 0.10), WS (Δ*n*_*D*_ ≈ 0.07) and NW (Δ*n*_*D*_ ≈ 0.07) network, indicating that the scale free networks are more control vulnerable than both the ER random and small world networks, which is due to the existence of hub nodes resulting from the power-law degree distribution as shown in Fig 7b. Another notable finding is that though Liu et al. have shown that driver nodes tend to avoid to be hub nodes [15], here we show that attacking the hub nodes is still the most efficient way to harm the controllability of scale free networks.

The analytical result of *n*_*D*_ for the RA attack can be obtained by employing the cavity method [15]. The BA network follows a power-law degree distribution with *P*(*k*)∼2*m*^2^ *k*^−3^ [2], since the removal fraction *f* is small, we assume that the degree distribution *P*(*k*) is not significantly affected, which gives us [15]

$$\begin{array}{c} n_{D}∼\exp\left[-{\left\langle k\right\rangle }^{0}\left(1-f\right)\left(1-\frac{1}{\gamma^{0}-1}\right)\right] \end{array}$$ (27)

where 〈*k*〉^0^ and *γ*^0^ = −3 are the average degree and degree exponent of the original network, respectively. From Fig 6 we can see that the theoretical prediction agrees well with the numerical result with negligible difference.

**Fig 8:**
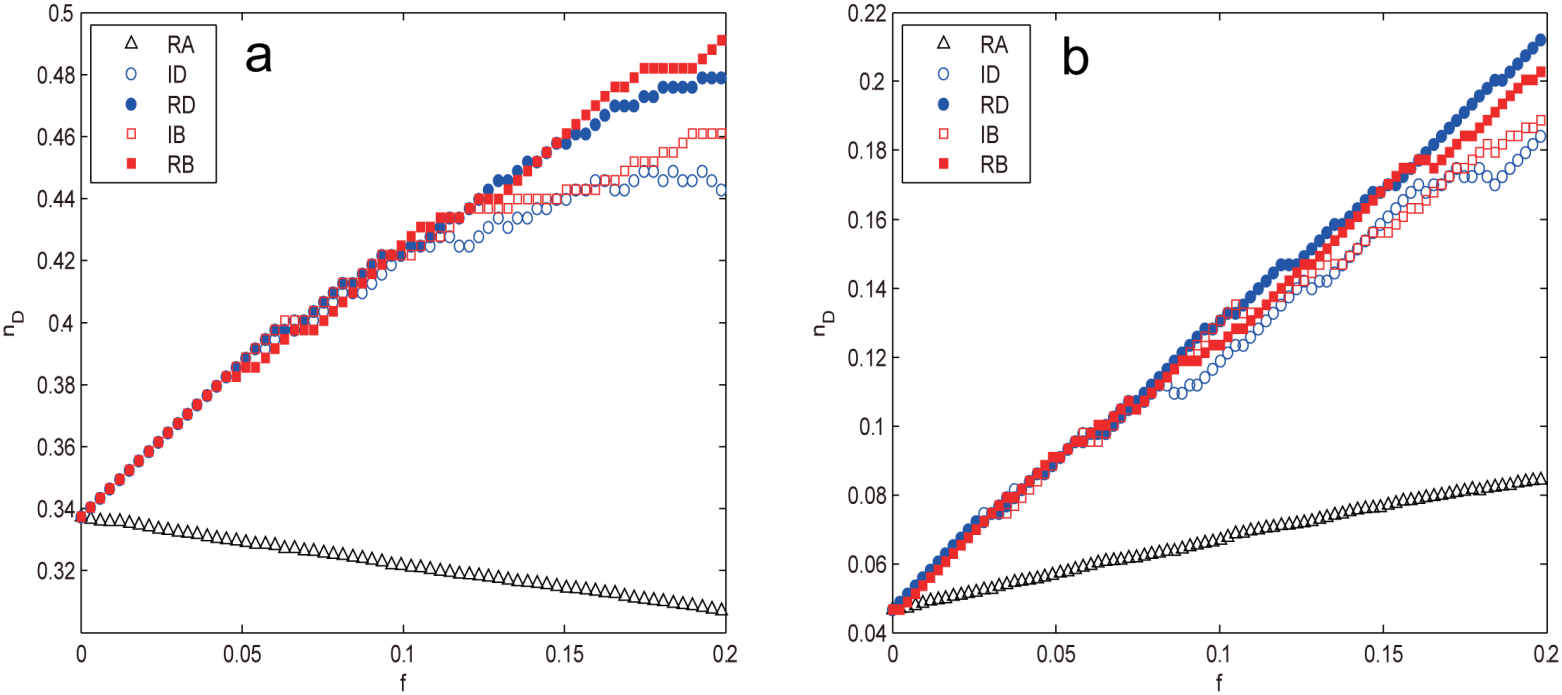
*n*_*D*_ as a function of removal fraction *f* under different node attacks for the (a) USAir97 and (b) Erdos971 network. The results are averaged over 100 independent runs.

**Fig 9:**
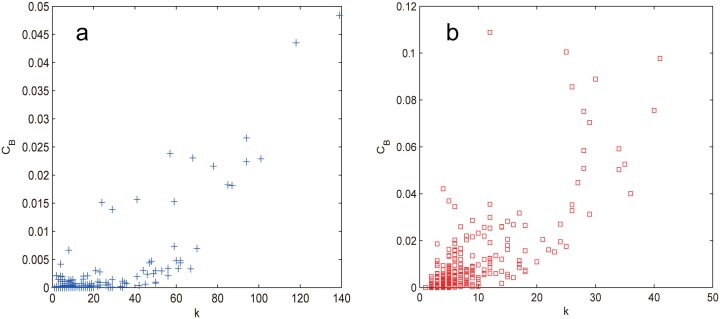
The node betweenness-degree correlation for the (a) USAir97 and (b) Erdos971 network.

The two real-world networks, USAir97 and Erdos971, display very similar vulnerable behaviors like the BA scale-free network for the intentional attacks as shown Fig 8, which is mainly due to their power-law degree distributions as shown Figs 1b and 2b. Nevertheless, the differences between them are significant. For USAir97, it can be seen that the intentional attacks harm the network controllability almost equally in the early stage of removal (f≲0.12), which is due to the strong betweenness-degree correlation in the region of high degrees as shown in Fig 9a, after that the strategies based on recalculated information prevail those based on initial configuration (RB, RD > IB, ID) owing to the redistribution of degree and betweenness information. Besides, the betweenness-based strategies outperform those degree-based ones (RB > RD and IB > ID) with very slight superiorities. The most confusing and unexpected behavior is that *n*_*D*_ decreases with *f* for RA, which means that we need less and less driver nodes to maintain the full control of network, i.e., the network controllability is indeed increasing, this abnormal phenomenon has not been observed in other cases and the reason behind is not clear and needs further research.

Erdos971 exhibits much similar behaviors like USAir97 as shown in Fig 8b. The intentional attacks ID, IB, RD and RB coincide in the early stage of removal (f≳0.08) due to the strong betweenness-degree correlation in the region of high degrees as shown in Fig 9b; these attacks also deviate in the last stage of removal (f≳0.17) in the approximate order RD > RB > IB > ID. The remarkable difference occurs in the interval 0.08≲f≲0.17, where RD prevails RB with less and less advantages and finally coincides with the latter at *f* ≈ 0.17, so does IB with ID. The recalculated strategies RD and RB are, as expected, more harmful than their counterparts ID and IB. Like the BA scale-free network, RD proves to be the most efficient way to harm the network’s controllability.

### Edge Attack

In this subsection, we study the attack vulnerability of network controllability against various edge attacks (see Sec. III for details of the edge attack strategies). Generally speaking, the edge attacks may be not as efficient as the node attacks since after removing *Ef* edges (*Nf* nodes), the former only removes *N*〈*k*〉*f*/2 edges whereas the latter can at most remove *Nf*〈*k*〉 edges. It should also be noted that the study presented here is different from that of Ref. [38], in which cascading edge failure is assumed and the removal of one edge may trigger cascading removals of other edges, here we do not make such assumption and the removal of one edge does not affect other edges’ removals at all.

**Fig 10:**
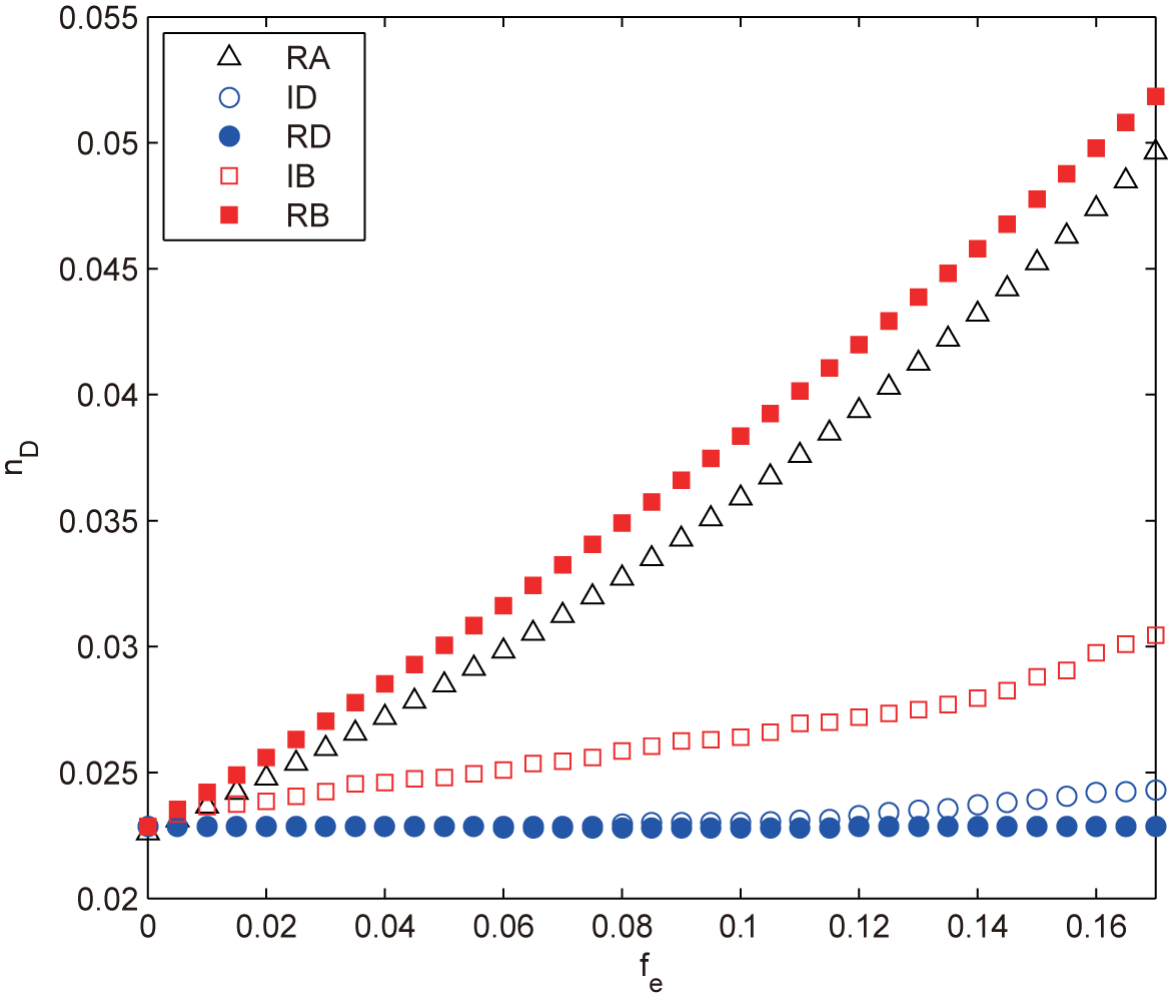
*n*_*D*_ as a function of the removal fraction *f*_*e*_ under different edge attacks for the ER random network. The results are averaged over 100 independent realizations.

Fig 10 shows the numerical results of the control robustness of the ER random network subject to edge strategies. We can see that the edge strategies are indeed less efficient than those node ones: Δ*n*_*D*_ ≈ 0.03 for the former compared with Δ*n*_*D*_ ≈ 0.11 for the latter (compare Figs 10 and 3). The RB procedure turns out to be the most destructive strategy, even so, it is just a little bit better than RA with very slight superiority (*n*_*D*_(RBA) − *n*_*D*_(RA) ≈ 0.003). *n*_*D*_s of others attacks (IB, ID and RD) are even inferior to that of RA with growing gaps, indicating that these strategies are even less efficient than random failures. Moreover, we can see that the degree-based attacks, ID and RD, can hardly harm the controllability at all as evident from the almost constant *n*_*D*_ shown in Fig 10.

These phenomena may seem difficult to understand at first glance, however, they can be explained by carefully analyzing the structural characteristics of ER. The ER random network has a short APL (∼ln *N*/ln〈*k*〉) due to its high proportion of shortcuts, such edges are removed by RB for their high betweenness and also have a great chance to be removed by RA. Therefore both RB and RA remove almost the same set of edges, resulting in almost the same damage to network controllability. This result may suggest that the distance measures like edge betweenness also affect the networks controllability. Given the importance of shortcuts in small-world networks, we infer that the edge betweenness-based strategies IB and RB, especially RB, will be more harmful in WS and NW. In addition, the difference between RB and IB is caused by the redistribution of edge betweenness, which is easy to understand, since as the removals proceed, the less shortcuts have to bear the same amount of shortest paths.

**Fig 11:**
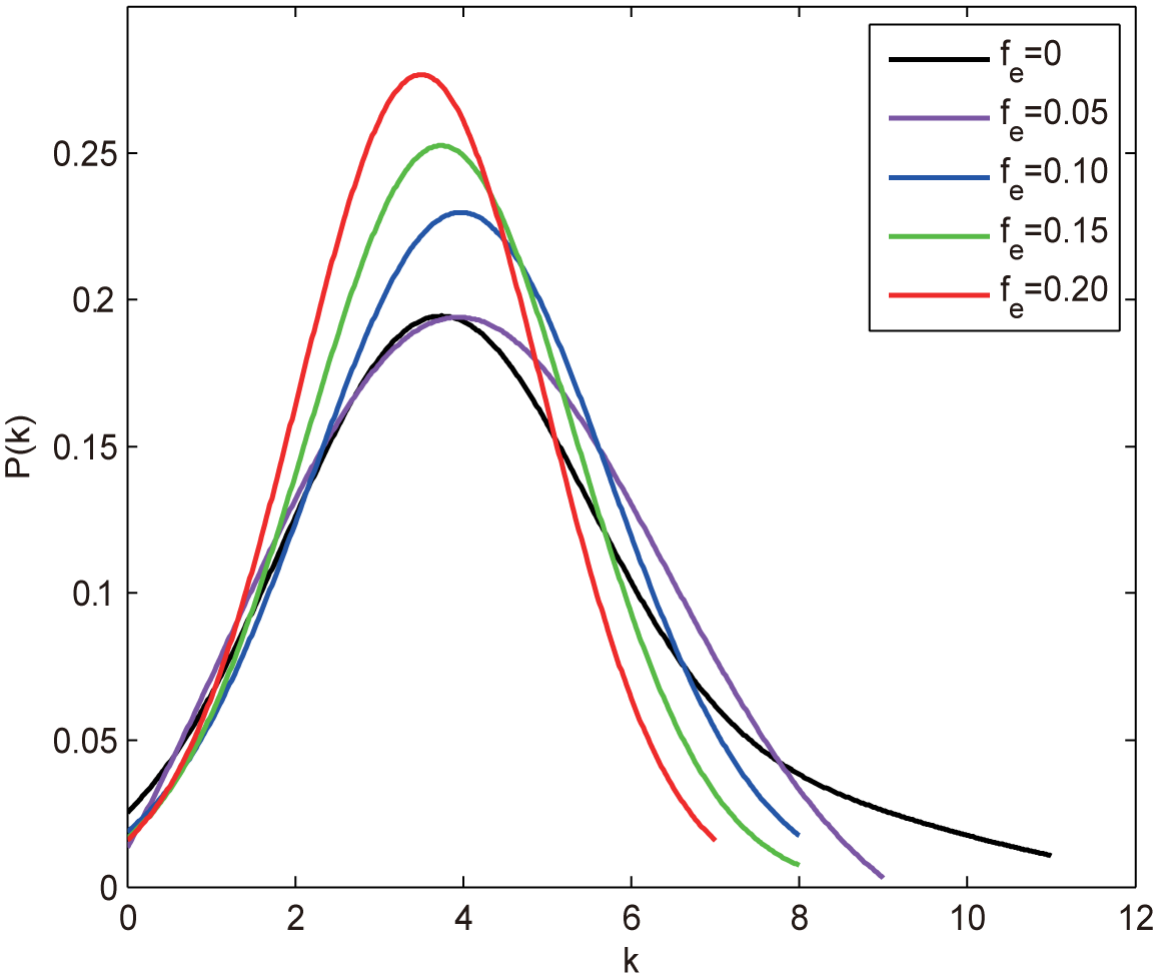
The degree distribution *P*(*k*) of the ER random network under different edge removal fraction *f*_*e*_ subject to the ID attack. *f*_*e*_ = 0 denotes the initial degree distribution.

The degree-based attacks, ID and RD, tend to preferentially remove edges that connect two high degree nodes (Eq (10)), which will weaken the degree of hub nodes and thus increase the number of medium degree nodes, resulting in a more centralized degree distribution as confirmed in Fig 11. This implies that the more homogeneous network may contribute to the results. To confirm this observation, we define the network heterogeneity as the standard deviation of degree distribution, i.e., *H* = [∑(*k*_*i*_−〈*k*〉)^2^/*N*]^1/2^. We then calculate the average degree 〈*k*〉, the degree heterogeneity *H* and the average betweenness centrality 〈*B*〉 under different *f*_*e*_ subject to ID attack and IB attack (as a comparison), the results are shown in Table 2. As 〈*k*〉 behaves identically for all the edge attacks, it does not require much elaboration. From Table 2, we can see that *H* decays with *f*_*e*_ whereas 〈*B*〉 increases with *f*_*e*_ for ID attack. Note that 〈*B*〉 exhibits almost the same behaviors for IB attack, which excludes the impact of 〈*B*〉 on the experimental results. In contrast, the declining speed of *H* for ID attack is much faster than that for IB attack, suggesting the homogeneous degree distribution is the key factor that contributes to such results. Liu et al. have pointed out that the degree heterogeneity affects network controllability and the homogeneous networks are easier to control [15]. As the degree-based attacks always make the network more homogeneous and thus easier to control (smaller *n*_*D*_), we infer that the two strategies are not efficient to attack the network controllability, especially for scale-free networks with biased degree structure. One thing to note is that the edge-based attacks also make 〈*k*〉 smaller, resulting in larger *n*_*D*_ according to Eq (3), which compensates for the decrease of *n*_*D*_, thus *n*_*D*_ stays almost unchanged.

**Table 2:** The structural characteristics of ER random network, including average degree 〈*k*〉, degree heterogeneity *H* and average betweenness centrality 〈*B*〉, vary with *f*_*e*_ subject to ID and IB attacks.

*f*_*e*_	0%	5%	10%	15%	20%
〈*k*〉_ID_	4.0	3.8	3.6	3.4	3.2
*H*_ID_	2.24	1.87	1.64	1.52	1.38
〈*B*〉_ID_	0.013	0.014	0.015	0.016	0.017
〈*k*〉_IB_	4.0	3.8	3.6	3.4	3.2
*H*_IB_	2.24	2.02	1.84	1.73	1.61
〈*B*〉_IB_	0.013	0.014	0.014	0.016	0.017

**Fig 12:**
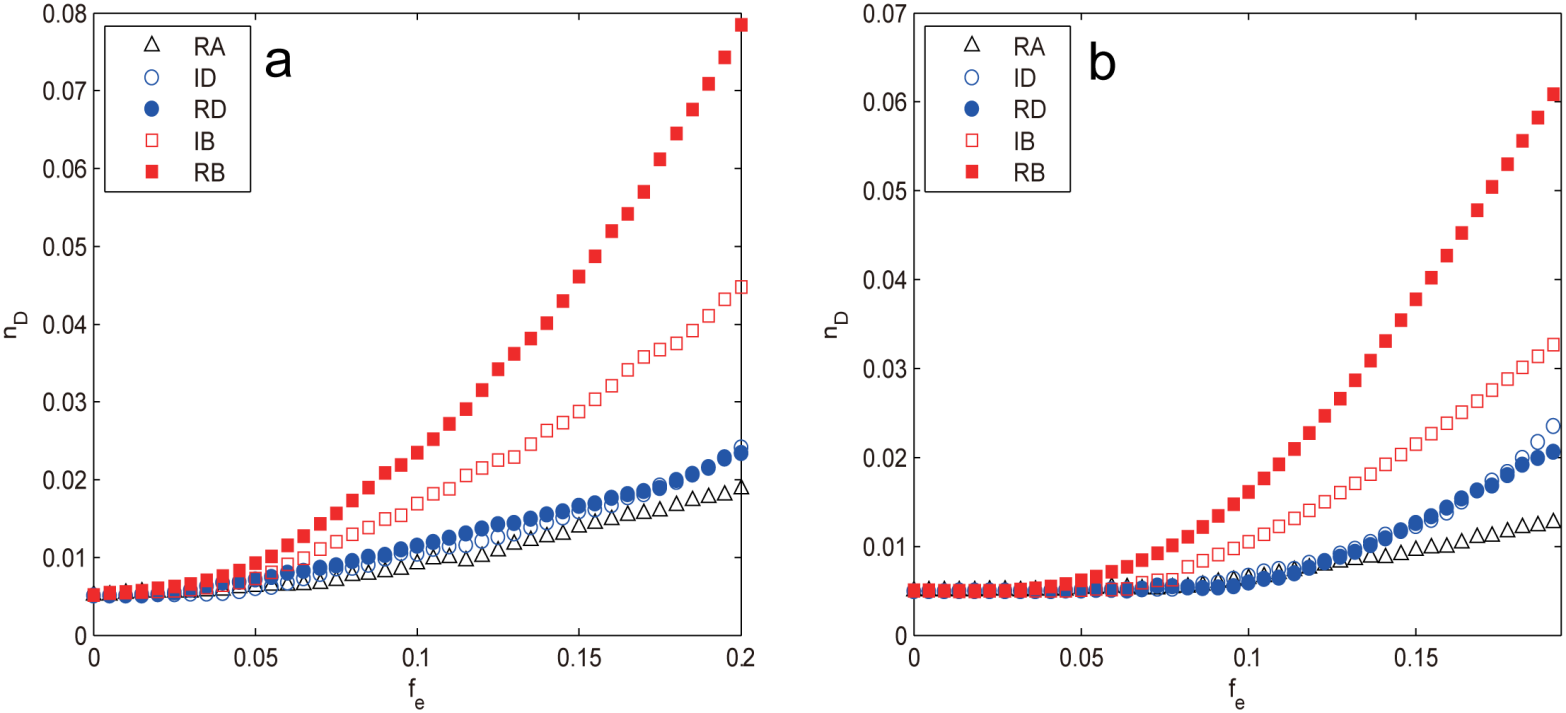
*n*_*D*_ as a function of removal fraction *f*_*e*_ subject to different edge attacks for (a) WS network and (b) NW network. The results are averaged over 100 independent realizations.

The controllability of two small-world networks, WS and NW, again displays quite similar vulnerability against edge attacks as shown in Fig 12. *n*_*D*_ retains almost constant in the early stage of attack ($f_{e}≲0.05$ for WS and $f_{e}≲0.07$ for NW), indicating that the network controllability is almost not affected, however, after that the attacks harm the network controllability in the order RB > IB > ID ≈ RD > RA. The betweenness-based strategies prove to be the most efficient way to damage the network controllability, which lives up to our previous conjecture. The reason for this is that the shortcuts in small-world networks play an important role in forming the so-called “small world” phenomenon, which shorten the distance between nodes that would otherwise be much farther. As such, these shortcuts usually have higher edge betweenness than ordinary edges and become the prior targets of the betweenness-based attacks. The removals of such shortcuts make the network lose most resemblance to the original topology and quickly degenerate into the one dimensional regular lattice with much larger APL, making the network harder to control. The difference between RB and IB is due to the redistribution of edge betweenness information: the less shortcuts have to bear the same number of shortest paths as the removals proceed. It should be noted that though RB and IB attack the same set of shortcuts, RB is still more efficient than IB, suggesting that the distance-based measures like edge betweenness indeed affect network’s controllability.

**Fig 13:**
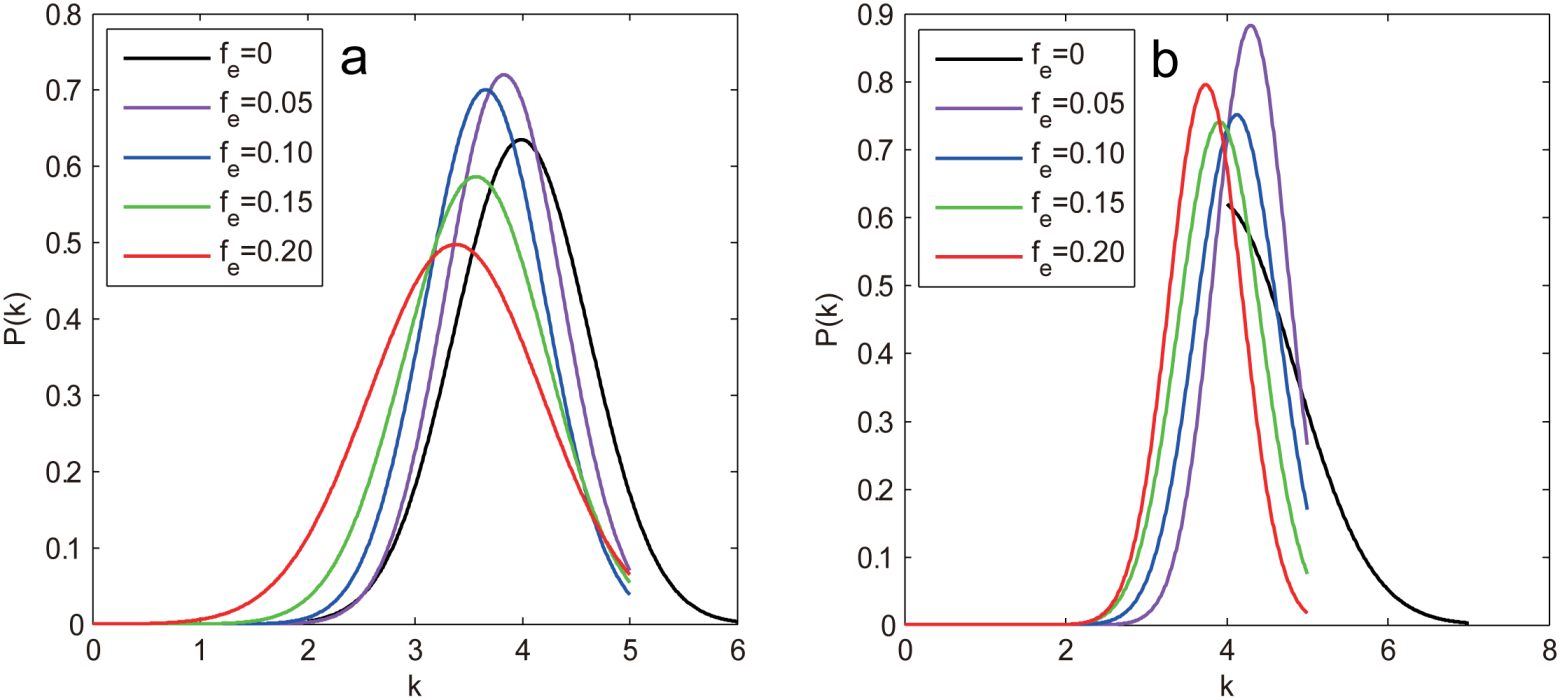
The degree distribution under different *f*_*e*_ subject to the ID attack for (a) WS network and (b) NW network.

In contrast, the two edge degree-based attacks are much less harmful than the betweenness-based ones and just comparable to random attack as seen in Fig 12. This is mainly because the degree-based strategies make the network more homogeneous and thus easier to control as has been analyzed previously. However, the real situation is slightly different. In Fig 13, we plot the degree distribution under different *f*_*e*_ subject to IDA for both networks, it can be seen that the peak of the curve continues to shift to left as the removal goes on, revealing that the network average degree is decreasing, while the network heterogeneity seems to be increasing judging from the more dispersed degree distribution. The more accurate numerical results in Table 3 show that the network heterogeneity, in fact, first decreases for small *f*_*e*_ ($f_{e}≲0.05$ for WS and $f_{e}≲0.07$ for NW) then increases until the end. This explains the behavior of *n*_*D*_: for small *f*_*e*_ it stays constant due to the opposite effects of 〈*k*〉 and *H*, after that it starts to increase quickly as both factors make the network more difficult to control.

**Table 3:** The average degree 〈*k*〉 and degree heterogeneity *H* under different *f*_*e*_ subject to IDA for WS and NW network.

*f*	0%	5%	10%	15%	20%
〈*k*〉(WS)	4.00	3.80	3.60	3.40	3.20
*H*(WS)	0.640	0.600	0.671	0.762	0.819
〈*k*〉(NW)	4.40	4.20	4.00	3.80	3.60
*H*(NW)	0.662	0.492	0.671	0.755	0.809

**Fig 14:**
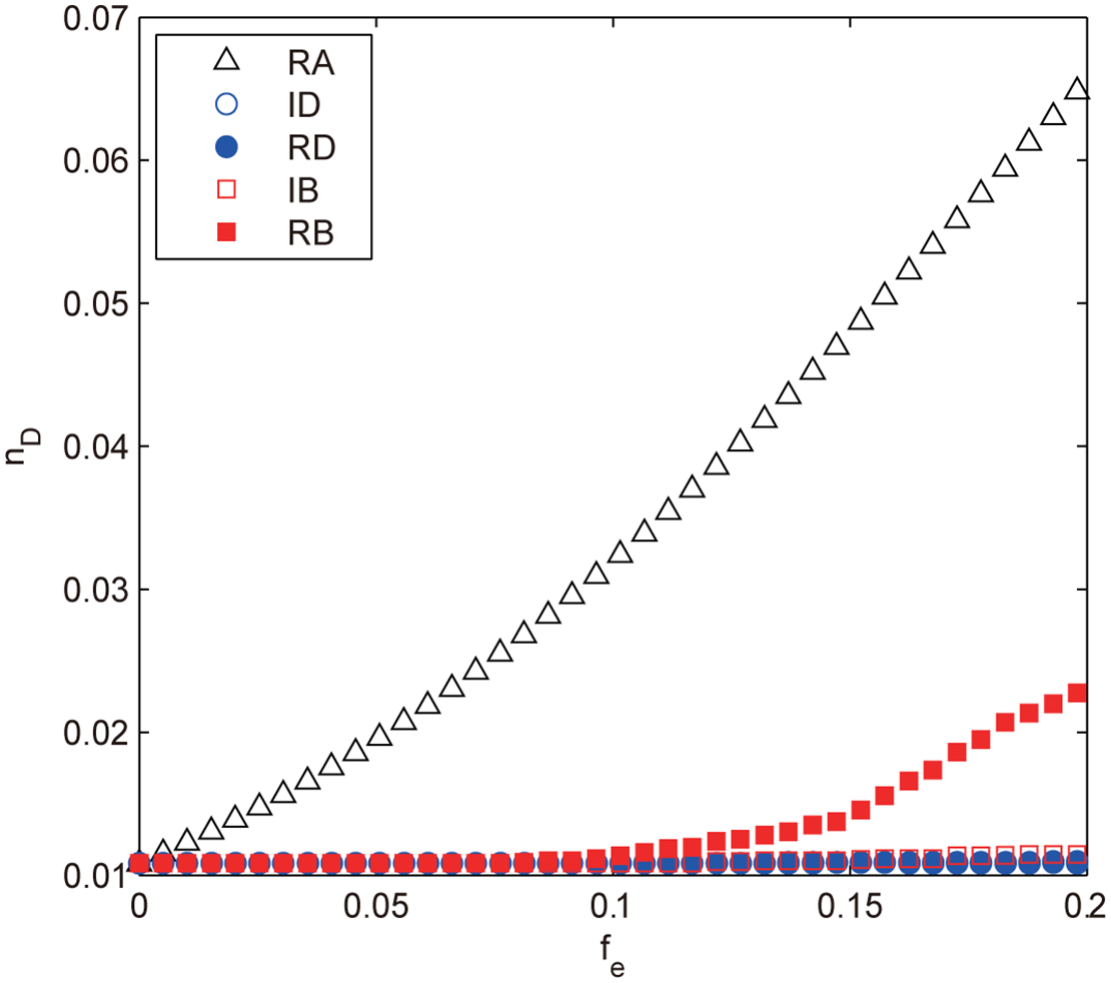
*n*_*D*_ as a function of *f*_*e*_ under different edge attacks for the BA scale-free network. The results are averaged over 100 independent realizations.

**Fig 15:**
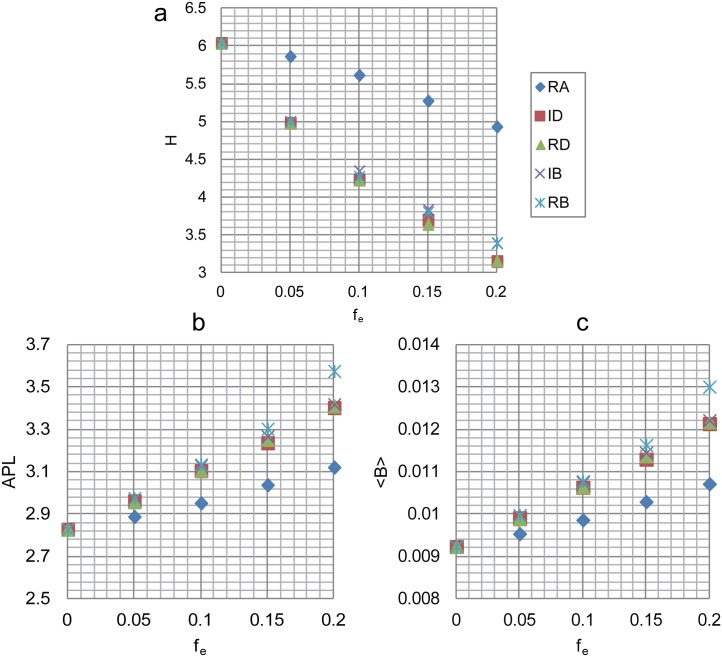
The structural characteristics of BA network vary with the edge removal fraction *f*_*e*_. The characteristics include (**a**) the degree heterogeneity *H*, (**b**) APL and (**c**) the average betweenness centrality 〈*B*〉.

Compared with the node attacks, the BA scale-free network for edge attacks exhibits strikingly different behaviors as shown in Fig 14. The random attack proves to be the most harmful strategy whereas the other attacks are far less efficient with broad range coincidence: *n*_*D*_ for ID, RD and IB stays constant throughout the removal; *n*_*D*_ for RB also changes little for $f_{e}≲0.1$, thereafter it starts to increase, but the growth is quite insignificant (Δ*n*_*D*_ ≈ 0.012) compared with that of RA. Since all the strategies affect the network degree equally, the difference must result from other factors. In Fig 15, we explore the variations of related structural characteristics of the BA scale-free network as the removal procedure proceeds. It can be seen that the network heterogeneity *H* under the deliberate attacks decays much faster than that of RA, which is easy to understand since both the degree-based and betweenness-based strategies preferentially remove edges that connect hub nodes, which weakens the high degree nodes and makes the network become more homogeneous and thus easier to control (smaller *n*_*D*_), resulting in the inferior performance of the deliberate attacks compared with that of RA. The difference between RB and the other deliberate attacks can be attributed to the distance-based measures as shown in Fig 15b and 15c, where both the APL and the average betweenness centrality 〈*B*〉 for RB prevail other attacks with increasing advantages for $f_{e}≳0.1$, which also explains the sudden increase of its *n*_*D*_ at *f*_*e*_ ≈ 0.1.

**Fig 16:**
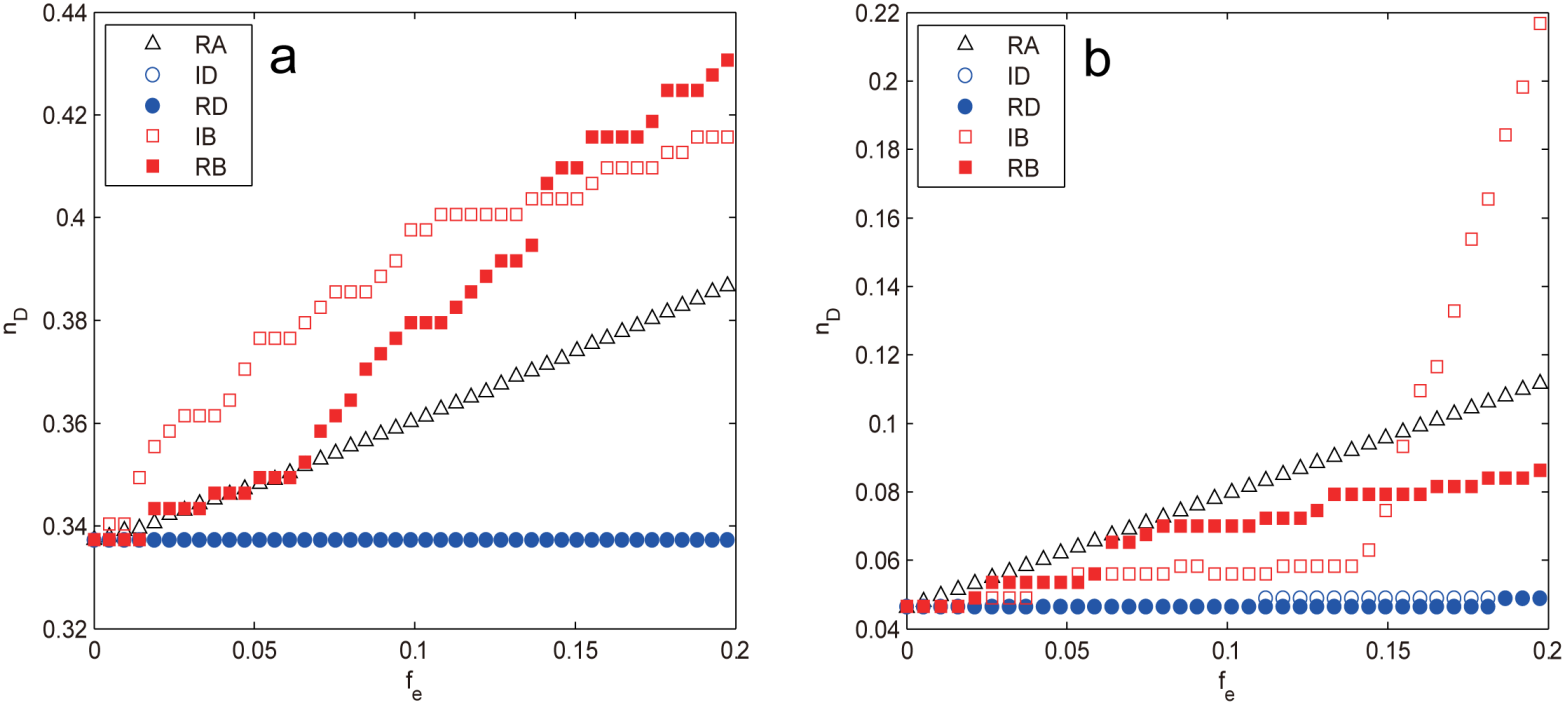
*n*_*D*_ as a function of *f*_*e*_ subject to different edge attacks for (a) USAir97 network and (b) Erdos971 network. The results are averaged over 100 independent realizations.

The two real-world networks, USAir97 and Erdos971, display again quite similar control vulnerability under edge attacks as shown in Fig 16. The degree-based strategies still have little effects on *n*_*D*_ whereas RA continues to harm the controllability steadily as usual. The notable difference is the behaviors of two betweenness-based strategies. For USAir97, both IB and RB prevail RA with more and more advantages; while for Erdos971, the order is reversed—RA remains prevailing over IB and RB for $f_{e}≲0.15$, thereafter IB starts to damage the controllability drastically and becomes the most harmful strategy. Once again these behaviors can be mainly attributed to the distanced-based measures like betweenness centrality. Nevertheless, the behaviors cannot match any of the model networks, implying that there may be other factors contributing to the network controllability.

### More Results

Despite that the canonical model networks can reveal general concepts of control robustness, our ultimate goal is to understand the control robustness of real-world networks. Therefore, we further investigate the attack vulnerability of network controllability for 25 real networks subject to different node and edge attacks using the same strategies, the results are shown in Tables 4 and 5.

**Table 4:** The node attack vulnerability of the real networks analyzed in this paper.

Type	Name	*N*	*L*	*n*_*D*_	*n*_*D*_ under node attack				
					RA	IDA	RDA	IBA	RBA
Regulatory	TRN-Yeast-1	4,441	12,873	0.965	0.869 [0.771]	0.897 [0.798]	0.900 [0.800]	0.894 [0.794]	0.886 [0.787]
	TRN-Yeast-2	688	1,079	0.821	0.738 [0.660]	0.815 [0.765]	0.823 [0.801]	0.773 [0.692]	0.769 [0.689]
	TRN-EC-2	418	519	0.751	0.677 [0.612]	0.739 [0.691]	0.754 [0.754]	0.722 [0.639]	0.718 [0.636]
	Ownership-USCrop	7,253	6,726	0.820	0.750 [0.680]	0.812 [0.731]	0.832 [0.800]	0.788 [0.688]	0.775 [0.675]
Trust	College student	32	96	0.188	0.188 [0.250]	0.188 [0.219]	0.219 [0.250]	0.219 [0.250]	0.219 [0.281]
	Prison inmate	67	182	0.134	0.164 [0.164]	0.149 [0.179]	0.164 [0.194]	0.164 [0.179]	0.164 [0.224]
	WikiVote	7,115	103,689	0.666	0.600 [0.534]	0.668 [0.663]	0.664 [0.662]	0.677 [0.672]	0.678 [0.658]
Food web	Ythan	135	601	0.511	0.467 [0.430]	0.563 [0.570]	0.556 [0.570]	0.548 [0.563]	0.563 [0.570]
	Little Rock	183	2,494	0.541	0.497 [0.454]	0.541 [0.541]	0.541 [0.541]	0.541 [0.541]	0.546 [0.546]
	Grassland	88	137	0.523	0.477 [0.409]	0.534 [0.580]	0.534 [0.580]	0.545 [0.545]	0.557 [0.602]
	Seagrass	49	226	0.265	0.265 [0.245]	0.286 [0.265]	0.265 [0.265]	0.286 [0.265]	0.265 [0.265]
Metabolic	*E. coli*	2,275	5,763	0.382	0.367 [0.346]	0.457 [0.424]	0.480 [0.581]	0.378 [0.350]	0.481 [0.578]
	*S. cerevisiae*	1,511	3,833	0.329	0.325 [0.312]	0.406 [0.365]	0.428 [0.527]	0.326 [0.311]	0.428 [0.525]
	*C. elegans*	1,809	1,303	0.657	0.603 [0.545]	0.641 [0.612]	0.655 [0.654]	0.655 [0.655]	0.655 [0.655]
Electronic	s838	512	819	0.232	0.246 [0.244]	0.313 [0.305]	0.314 [0.354]	0.254 [0.275]	0.311 [0.367]
circuits	s420	252	399	0.234	0.262 [0.274]	0.310 [0.313]	0.310 [0.353]	0.262 [0.274]	0.310 [0.369]
	s208	122	189	0.238	0.230 [0.262]	0.303 [0.328]	0.303 [0.352]	0.279 [0.287]	0.311 [0.361]
Neuronal	*C. elegans*	297	2,345	0.165	0.158 [0.152]	0.192 [0.219]	0.185 [0.222]	0.178 [0.205]	0.195 [0.242]
WWW	Political blogs	1,224	19,025	0.356	0.334 [0.305]	0.408 [0.456]	0.408 [0.454]	0.417 [0.470]	0.425 [0.475]
Internet	p2p-1	10,876	39,994	0.552	0.503 [0.454]	0.571 [0.571]	0.576 [0.581]	0.573 [0.576]	0.575 [0.590]
	p2p-2	8,846	31,839	0.578	0.525 [0.473]	0.585 [0.579]	0.590 [0.595]	0.589 [0.591]	0.595 [0.605]
	p2p-3	8,717	31,525	0.577	0.527 [0.474]	0.585 [0.583]	0.588 [0.592]	0.591 [0.593]	0.596 [0.604]
Organizational	Consulting	46	879	0.043	0.043 [0.043]	0.043 [0.043]	0.043 [0.043]	0.043 [0.043]	0.043 [0.043]
	Manufacturing	77	2,228	0.013	0.013 [0.013]	0.013 [0.013]	0.013 [0.013]	0.013 [0.013]	0.013 [0.013]
	University	81	817	0.012	0.025 [0.025]	0.012 [0.025]	0.012 [0.025]	0.025 [0.037]	0.012 [0.025]

For each network, we show its type, name, number of nodes (*N*), number of edges (*L*), the initial density of driver nodes (*n*_*D*_) and *n*_*D*_ after 10% [20%] nodes are removed. All the networks are directed and *n*_*D*_ is calculated with the structural controllability framework [15]. For networks with *N* ≥ 3000, nodal betweenness centrality is estimated with approximate algorithm [47] to speed up the computation. For data sources and references, see S1 Table.

**Table 5:** The edge attack vulnerability of real networks analyzed in this paper.

Type	Name	*N*	*L*	*n*_*D*_	*n*_*D*_ under edge attack				
					RA	IDA	RDA	IBA	RBA
Regulatory	TRN-Yeast-1	4,441	12,873	0.965	0.965 [0.965]	0.965 [0.965]	0.965 [0.965]	0.965 [0.965]	0.965 [0.965]
	TRN-Yeast-2	688	1,079	0.821	0.826 [0.830]	0.821 [0.821]	0.821 [0.823]	0.830 [0.846]	0.830 [0.833]
	TRN-EC-2	418	519	0.751	0.758 [0.778]	0.751 [0.751]	0.751 [0.751]	0.768 [0.778]	0.773 [0.785]
	Ownership-USCrop	7,253	6,726	0.820	0.833 [0.843]	0.820 [0.821]	0.820 [0.820]	0.832 [0.849]	0.842 [0.852]
Trust	College student	32	96	0.188	0.188 [0.188]	0.188 [0.188]	0.188 [0.188]	0.219 [0.313]	0.250 [0.281]
	Prison inmate	67	182	0.134	0.164 [0.209]	0.134 [0.149]	0.134 [0.134]	0.179 [0.224]	0.164 [0.224]
	WikiVote	7,115	103,689	0.666	0.667 [0.669]	0.666 [0.666]	0.666 [0.666]	0.674 [0.678]	0.666 [0.676]
Food web	Ythan	135	601	0.511	0.533 [0.533]	0.511 [0.511]	0.511 [0.511]	0.541 [0.548]	0.533 [0.600]
	Little Rock	183	2,494	0.541	0.541 [0.541]	0.541 [0.541]	0.541 [0.541]	0.557 [0.579]	0.568 [0.601]
	Grassland	88	137	0.523	0.545 [0.591]	0.523 [0.523]	0.523 [0.523]	0.557 [0.602]	0.580 [0.659]
	Seagrass	49	226	0.265	0.265 [0.286]	0.265 [0.286]	0.265 [0.265]	0.306 [0.347]	0.327 [0.327]
Metabolic	*E. coli*	2,275	5,763	0.382	0.406 [0.436]	0.383 [0.385]	0.382 [0.383]	0.390 [0.410]	0.394 [0.410]
	*S. cerevisiae*	1,511	3,833	0.329	0.355 [0.392]	0.330 [0.333]	0.329 [0.330]	0.343 [0.351]	0.343 [0.361]
	*C. elegans*	1,809	1,303	0.657	0.666 [0.689]	0.657 [0.657]	0.657 [0.657]	0.669 [0.684]	0.669 [0.685]
Electronic	s838	512	819	0.232	0.285 [0.340]	0.246 [0.264]	0.232 [0.246]	0.299 [0.334]	0.346 [0.422]
circuits	s420	252	399	0.234	0.294 [0.333]	0.246 [0.274]	0.234 [0.242]	0.302 [0.341]	0.353 [0.425]
	s208	122	189	0.238	0.262 [0.320]	0.238 [0.262]	0.238 [0.238]	0.295 [0.361]	0.352 [0.434]
Neuronal	*C. elegans*	297	2,345	0.165	0.172 [0.178]	0.165 [0.165]	0.165 [0.165]	0.256 [0.269]	0.205 [0.273]
WWW	Political blogs	1,224	19,025	0.356	0.373 [0.389]	0.356 [0.356]	0.356 [0.356]	0.431 [0.468]	0.404 [0.494]
Internet	p2p-1	10,876	39,994	0.552	0.558 [0.566]	0.556 [0.562]	0.552 [0.553]	0.558 [0.564]	0.565 [0.573]
	p2p-2	8,846	31,839	0.578	0.585 [0.591]	0.582 [0.585]	0.578 [0.578]	0.583 [0.588]	0.588 [0.597]
	p2p-3	8,717	31,525	0.577	0.582 [0.589]	0.583 [0.586]	0.578 [0.578]	0.583 [0.587]	0.589 [0.597]
Organizational	Consulting	46	879	0.043	0.043 [0.043]	0.043 [0.043]	0.043 [0.043]	0.065 [0.087]	0.130 [0.174]
	Manufacturing	77	2,228	0.013	0.013 [0.013]	0.013 [0.013]	0.013 [0.013]	0.013 [0.026]	0.052 [0.065]
	University	81	817	0.012	0.012 [0.012]	0.012 [0.012]	0.012 [0.012]	0.062 [0.074]	0.074 [0.099]

For each network, we show its type, name, number of nodes (*N*), number of edges (*L*), the initial density of driver nodes (*n*_*D*_) and *n*_*D*_ after 10% [20%] edges are removed. All the networks are directed and *n*_*D*_ is calculated with the structural controllability framework [15]. For networks with *N* ≥ 3000, nodal betweenness centrality is estimated with approximate algorithm [47] to speed up the computation. For data sources and references, see S1 Table.

From Table 4, we can see that the regulatory networks such as the transcriptional regulatory network of *Saccharomyces cerevisiae* (TRN-Yeast-1 [48], TRN-Yeast-2 [49]) and *Echerichia coli* (TRN-EC-2 [49]) and the ownership network of US telecommunications and media corporations (Ownership-USCrop [50]) exhibit strikingly control robust under attacks: *n*_*D*_ decreases instead of increases throughout the removal for all the five strategies, meaning that we need ever less and less driver nodes to control the whole network and the network controllability is, in fact, increasing. This phenomenon has not been observed before our work and clearly shows that the control robustness of regulatory networks are more than good enough. The other class of networks with great control robustness is the case of the intra-organizational networks [51, 52], in which *n*_*D*_ stays constant for all five attacks, indicating that the attacking cannot affect the network controllability at all. One exception is the university network [52], which can only resist some attacks (ID, RD, RB) for small *f*_*e*_ ≤ 10%. The control robustness of other networks can be, more or less, grasped from the model networks. The betweenness-based attacks and the degree-based attacks are almost equal harmful for the food web networks [53–55] and the metabolic networks [56]. However, for most other networks, the former are usually more harmful than the latter and so are the strategies based on recalculated information than those based on initial information. The last interesting observation is that most of the real-world networks seem to have a decreasing or constant *n*_*D*_ for RA, indicating that the real-world networks tend to be control robust against random node failures.

In Table 5, we display the attack vulnerability of real-world networks subject to edge attacks. It can be seen that the edge degree-based attacks (both ID and RD) still cannot damage the network controllability at all as observed in the model networks, which, as has been explained, is because of the fact that such strategies make networks become more homogenous and thus easier to control. Our conclusion is that the edge degree defined as Eq (10) is not a good quantity to measure the importance of an edge in terms of network controllability. Another significant difference is the behaviors of the random edge failures. Here only the intra-organizational networks [51, 52] show good control robustness against random edge failures while other networks do not. The rest cases are very similar. In general, RB is the most harmful strategy, followed by IB, and RA is the least one. Two exceptions are the trust networks [57–59] in which IB are more efficient than RB and the metabolic networks [56] in which RA suppresses both IB and RB with slight advantage.

In summary, the real world networks display rather diverse control robust behaviors, which may suggest that one should really choose the attack strategies carefully before harming the controllability of real networks as it may affect the results to a very large extent.

## Summary and Conclusions

In this paper, we have systematically investigate the attack vulnerability of network controllability for the canonical model networks as well as the real-world networks subject to five different strategies on the basis of nodes and edges. The strategies are chosen based on degree and betweenness centralities evaluated with the initial information as well as the recalculated information, among which random failure is as a comparison. We found that for node attacks, the ER random network is more control vulnerable to the degree-based attacks (RD and ID) with Δ*n*_*D*_ ≈ 0.12 while the small-world networks (WS and NW) are more vulnerable to the betweenness-based attack (RB) with Δ*n*_*D*_ ≈ 0.07. The BA scale-free model network with an exponential degree distribution, which is one of the most important characteristics in real-world networks [60–63], turns out to be the most vulnerable network (Δ*n*_*D*_ ≈ 0.19) due to the existence of hub nodes and the high correlation between node degree and betweenness. The similar vulnerability is also observed in the two real-world networks, USAir97 and Erdos971. For edge attacks, the strategies are not as that efficient as the node-based ones. For example, both RD and ID can hardly harm the controllability at all for all the tested networks, and even the most harmful strategy RB only damages the ER random network with Δ*n*_*D*_ ≈ 0.033 and the small-world networks with Δ*n*_*D*_ ≈ 0.065. The notable difference is the BA scale-free network, which, out of our expectation, exhibits great control robustness to all the intentional attacks with almost constant *n*_*D*_. The two real-world networks, USAir97 and Erdos971, display complicated behaviors different from any of the model networks with Δ*n*_*D*_ ≈ 0.09 for the former and Δ*n*_*D*_ ≈ 0.18 for the latter, which suggests that there may be other factors contributing to the controllability.

We also investigate the control robustness of 25 real-world networks and find that the control vulnerability of real-world networks is much different from that of model networks. For example, most of the real-world networks exhibit good control robustness against random node failures, which are not observed in the model networks. The regulatory networks are even excessively robust to all the node attacks with decreasing *n*_*D*_s (Δ*n*_*D*_ ≤ 0), followed by the intra-organizational networks with constant *n*_*D*_s (Δ*n*_*D*_ = 0). For other networks subject to node attacks, the betweenness-based strategies are more harmful than the degree-based ones, and so are the strategies based recalculated information than their counterparts. In contrast, for edge attacks, only the intra-organizational networks are robust to random edge failures whereas other networks are harmed by the attacks in the order RB > IB > RA. In addition, we find that edge degree is not a good quantity to measure the importance of an edge in terms of network controllability.

Our results raise several questions to answer to help us better understand the attack vulnerability of network controllability. For example, we have shown that besides the degree distribution, there are other factors affecting the network controllability, what are the factors and how do they affect the controllability? Besides, the real-world networks usually have certain defence against attacks and failures, so how about the attack vulnerability of network controllability with defence?

## Supporting Information

S1 Table Real networks analyzed in this paper. (PDF)
