# Supplementary material for: Attack Vulnerability of Network Controllability
Source: PLoS One. 2016 Sep 2;11(9):e0162289. doi: 10.1371/journal.pone.0162289 (PMC5010274; doi:10.1371/journal.pone.0162289)
Supplement: S1 Table — (PDF) [file pone.0162289.s001.pdf]

Table S1: Real networks analyzed in this paper.

Type	Name	N	L	Description
Regulatory	TRN-Yeast-1 [1]	4,441	12,873	Transcriptional regulatory network of *S. cerevisiae*
	TRN-Yeast-2 [2]	688	1,079	Same as above (compiled by different group)
	TRN-EC-2 [2]	418	519	Transcriptional regulatory network of *E. coli*
	Ownership-USCrop [3]	7,253	6,726	Ownership network of US corporations
Trust	College student [4, 5]	32	96	Social networks of positive sentiment (college students)
	Prison inmate [4, 5]	67	182	Same as above (prison inmates)
	WikiVote [6]	7,115	103,689	Who-vote-whom network of Wikipedia users
Food web	Ythan [7]	135	601	Food Web in Ythan Estuary
	Little Rock [8]	183	2,494	Food Web in Little Rock lake
	Grassland [7]	88	137	Food Web in Grassland
	Seagrass [9]	49	226	Food Web in St. Marks Seagrass
Metabolic	*E. coli* [10]	2,275	5,763	Metabolic network of *E. coli*
	S. cerevisiae [10]	1,511	3,833	Metabolic network of *S. cerevisiae*
	C. elegans [10]	1,809	1,303	Metabolic network of *C. elegans*
Electronic	s838 [2]	512	819	Electronic sequential logic circuit
circuits	s420 [2]	252	399	Same as above
	s208 [2]	122	189	Same as above
Neuronal	*C. elegans* [11]	297	2,345	Neural network of *C. elegans*
WWW	Political blogs [12]	1,224	19,025	Hyperlinks between weblogs on US politics
Internet	p2p-1 [13, 14]	10,876	39,994	Gnutella peer-to-peer file sharing network
	p2p-2 [13, 14]	8,846	31,839	Same as above (at different time)
	p2p-3 [13, 14]	8,717	31,525	Same as above (at different time)
Organizational	Consulting [15]	46	879	Social network from a consulting company
	Manufacturing [15]	77	2,228	Social network from a manufacturing company
	University [16]	81	817	Social network among faculty members at a UK university

All the networks are directed; for each network, we show its type, name and reference; number of nodes (N) and edges (L); and brief description.

References

[1] Balaji S, Babu MM, Iyer LM, Luscombe NM, Aravind L. Comprehensive analysis of combinatorial regulation using the transcriptional regulatory network of yeast. Journal of molecular biology. 2006;360(1):213–227.

[2] Milo R, Shen-Orr S, Itzkovitz S, Kashtan N, Chklovskii D, Alon U. Network motifs: simple building blocks of complex networks. Science. 2002;298(5594):824–827.

[3] Norlen K, Lucas G, Gebbie M, Chuang J. EVA: Extraction, visualization and analysis of the telecommunications and media ownership network. In: Proceedings of International Telecommunications Society 14th Biennial Conference (ITS2002), Seoul Korea. Citeseer; 2002.

[4] Milo R, Itzkovitz S, Kashtan N, Levitt R, Shen-Orr S, Ayzenshtat I, et al. Superfamilies of evolved and designed networks. Science. 2004;303(5663):1538–1542.

[5] Van Duijn MA, Zeggelink EP, Huisman M, Stokman FN, Wasseur FW. Evolution of sociology freshmen into a friendship network. Journal of Mathematical Sociology. 2003;27(2-3):153–191.

[6] Leskovec J, Huttenlocher D, Kleinberg J. Signed networks in social media. In: Proceedings of the SIGCHI Conference on Human Factors in Computing Systems. ACM; 2010. p. 1361–1370.

[7] Dunne JA, Williams RJ, Martinez ND. Food-web structure and network theory: the role of connectance and size. Proceedings of the National Academy of Sciences. 2002;99(20):12917–12922.

[8] Martinez ND. Artifacts or attributes? Effects of resolution on the Little Rock Lake food web. Ecological Monographs. 1991; p. 367–392.

[9] Christian RR, Luczkovich JJ. Organizing and understanding a winters seagrass foodweb network through effective trophic levels. Ecological Modelling. 1999;117(1):99–124.

[10] Jeong H, Tombor B, Albert R, Oltvai ZN, Barabási AL. The large-scale organization of metabolic networks. Nature. 2000;407(6804):651–654.

[11] Achacoso TB, Yamamoto WS. AY's Neuroanatomy of C. elegans for Computation. CRC Press; 1991.

[12] Adamic LA, Glance N. The political blogosphere and the 2004 US election: divided they blog. In: Proceedings of the 3rd international workshop on Link discovery. ACM; 2005. p. 36–43.

[13] Leskovec J, Kleinberg J, Faloutsos C. Graphs over time: Densification and shrinking diameters. In: ACM Transactions on Knowledge Discovery from Data (ACM TKDD. Citeseer;.

[14] Ripeanu M, Foster I, Iamnitchi A. Mapping the gnutella network: Properties of large-scale peer-to-peer systems and implications for system design. arXiv preprint cs/0209028. 2002;.

[15] Cross R, Parker A, Christensen CM, Anthony SD, Roth EA. The hidden power of social networks. Audio-Tech Business Book Summaries, Incorporated; 2004.

[16] Nepusz T, Petróczi A, Négyessy L, Bazsó F. Fuzzy communities and the concept of bridgeness in complex networks. Physical Review E. 2008;77(1):016107.
